# A novel Complex q-rung orthopair fuzzy Yager aggregation operators and their applications in environmental engineering

**DOI:** 10.1016/j.heliyon.2025.e41668

**Published:** 2025-01-03

**Authors:** Jabbar Ahmmad, Tahir Mahmood, Dragan Pamucar, Hafiz Muhammad Waqas

**Affiliations:** a SK-Research-Oxford Business College, Oxford, OX1 2EP, UK; b Department of Mathematics and Statistics, International Islamic University Islamabad, Pakistan; c Széchenyi István University, Győr, Hungary

**Keywords:** Cartesian form of complex q-rung orthopair fuzzy set, Temperature control systems, Environmental engineering, Yager aggregation operators

## Abstract

Improving human health and comfort in buildings requires efficient temperature regulation. Temperature control system has a significant contribution in minimizing the impact of climate change. Temperature control system is used in industry to control temperature. The polar form of complex Pythagorean fuzzy set is a limited notion because when decision makers take the value for membership degree as 0.71+ι0.81 then we can observe that the basic condition for complex Pythagorean fuzzy set fails to hold that is $r=\sqrt{0.71^{2}+0.81^{2}}=1.3661\notin \left[0,1\right]$. Moreover, we can observe that the Cartesian form of a complex Pythagorean fuzzy set is also a limited notion because it can never discus advance data. Hence keeping in mind these limitations of the existing notions, in this article, we have explored the Cartesian form of a complex q-rung orthopair fuzzy set. Moreover, we have developed the Yager operational laws based on a Cartesian form of complex q-rung orthopair fuzzy set. We have introduced aggregation theory named complex q-rung orthopair fuzzy Yager weighted average and complex q-rung orthopair fuzzy Yager weighted geometric aggregation operators in Cartesian form. Based on these aggregation operators, we have initiated a multi-attribute group decision-making (MAGDM) approach to define the reliability and authenticity of the developed theory. Furthermore, we have utilized this device algorithm in the selection of a temperature control system. The comparative study of the delivered approach shows the advancement and superiority of the delivered approach.

## 1 Introduction

To ensure ecological balance, promote sustainability, improve industrial operations, and support human health, temperature control systems (TCS) are essential. From urban areas to manufacturing plants and agricultural environments, these systems encompass a wide range of technologies and strategies aimed at regulating temperature in various natural and built settings. Effective control of temperature is required to deal with challenges due to climate change as heat waves, the occurrence of extreme weather events and increasing global temperatures, play а significant role. These controls could significantly save energy consumption; reduce greenhouse gas emissions and improve the efficiency of energy-intensive processes such as heating and cooling systems or during industrial-manufacturing use.

### 1.1 Significance of temperature control system

TCSs are important in environmental conservation by maximizing energy efficiency and reducing greenhouse gas emissions. They increase the efficiency of heating and cooling systems. In agriculture, accurate TC creates optimal planting conditions that minimize wastage while increasing crop yields. The significance of TCS is given by.

1. The TCS has a significant contribution to minimizing the impact of climate change by diminishing greenhouse gas releases. Solar panels and energy-efficient HVAC in the residential, commercial, and industrial sectors can help lower their carbon emissions. Systems such as these are contributing towards more efficient (e.g. through heat, cool and ventilation optimization) worldwide efforts to try to reduce the effects of climate change by reducing energy consumption.
2. Improving human health and comfort in buildings requires efficient temperature regulation. HVAC systems enhance indoor air quality and lower the incidence of heat-related diseases by regulating interior temperatures within tolerable ranges and making sure there is enough ventilation. By lowering temperatures in highly populated regions, these systems help lessen the impact of the urban heat island, improving public health and well-being.
3. TCSs are used in industry to keep temperatures constant for manufacturing processes or production operations. That high accuracy comes with higher energy efficiency, less waste and better product quality. This has a further benefit in supporting overall sustainability goals and operational efficiency through lower energy usage (and thus environmental impact cost), achieved via waste heat recovery systems to advanced cooling/heating technologies.
4. By streamlining combustion processes and cutting emissions, TCSs contribute significantly to pollution reduction. Cutting-edge industrial technologies increase combustion efficiency and reduce emissions of pollutants including Sulphur dioxide (SO2) and nitrogen oxides (NOx). Effective waste management techniques minimize environmental pollution and improve public health when waste incineration and treatment facilities maintain proper temperature control.
5. TCS also utilizes cutting-edge cooling and heating technologies. These advanced systems are designed to be highly efficient, using the least amount of energy possible while still maintaining the desired temperature. By optimizing energy usage, companies can reduce their carbon footprint and contribute to their sustainability goals.

### 1.2 Some resources to control temperature in the environment

Some resources and technologies are used to control temperature in various environmental settings.

**Smart Thermostats and Building Automation Systems:** Through automated temperature adjustments depending on occupancy and outside circumstances, smart thermostats and building automation systems maximize energy consumption. By making sure heating and cooling systems are adjusted based on actual demand, these technologies improve building energy efficiency. Building automation specialists like Siemens and Johnson Controls, as well as service providers like Nest and Eco bee, are good places to go for resources on smart thermostat technology.

**Fig. 1:**
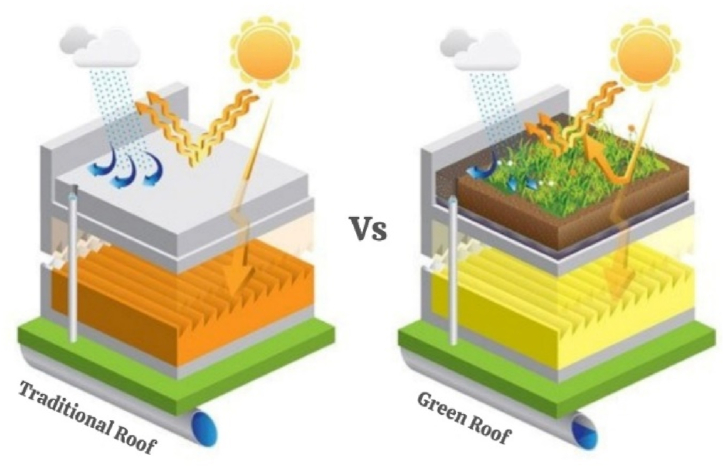
Green roof vs. traditional roof.

**Green Roofs and Walls:** Vegetation is used in green walls and roofs to control internal building temperatures, act as insulation, and lessen heat absorption. By strengthening natural cooling processes, they reduce urban heat island effects and save electricity. Research papers and case studies on green wall and roof technologies, as well as associations such as green building councils, provide resources for putting green infrastructure solutions into practice. The comparison between green roofs and traditional roofs is given in Fig. 1.

**Fig. 2:**
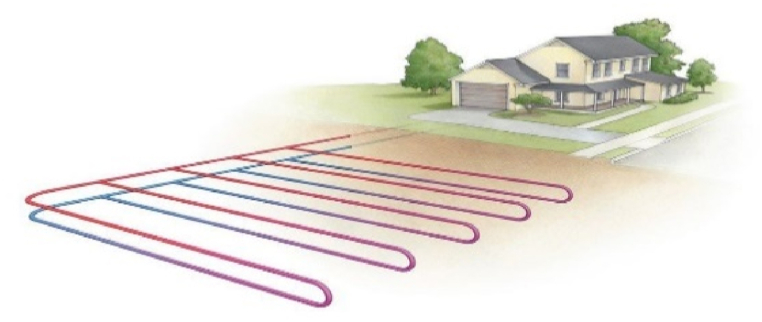
Geothermal heating and cooling system with Heating pump phenomena.

**Geothermal Heating and Cooling Systems:** Stable earth temperatures are used by geothermal systems to effectively heat and cool buildings. Compared to conventional HVAC systems, they provide environmentally friendly, low-maintenance heating and cooling options. Resources for geothermal heating and cooling technologies are available from the climate master and water furnace, two geothermal system vendors, as well as the international ground source heat pump association. The pictorial representation of the geothermal heating and cooling system is given in Fig. 2.

**Solar Shading and Window Treatments:** Window coverings and solar shading systems reduce solar heat gain and increase building energy efficiency. These innovations improve passenger comfort and lessen the demand for artificial cooling. Manufacturers of shading devices, suppliers of window coverings, and studies on energy-efficient building materials and passive solar architecture are some of the resources for solar shading and window treatments.

**Waste Heat Recovery Systems:** Systems for recovering waste heat from industrial operations are used to recover and repurpose heat for power generation or heating. These systems use waste heat that would otherwise be released into the atmosphere to increase energy efficiency and lower greenhouse gas emissions. Publications on waste heat utilization, energy management firms, and research institutes specializing in industrial efficiency are good sources of information about waste heat recovery technology.

**Fig. 3:**
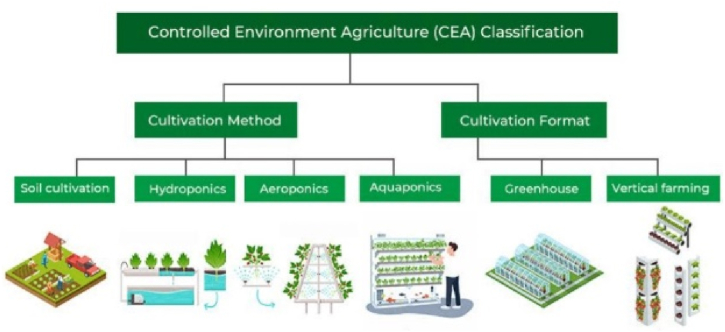
Controlled Environment Agriculture Classification analysis.

**Climate-Controlled Agriculture (Greenhouses, Vertical Farms):** Climate-controlled agricultural systems adjust humidity, temperature, and other environmental elements to maximize crop development in under-regulated settings. TCS is utilized by greenhouses and vertical farms to prolong their growth seasons, enhance agricultural yields, and preserve resources. Providers of greenhouse technology, societies devoted to sustainable agriculture and vertical farming, and agricultural research institutes are some of the resources available for climate-controlled agriculture. Fig. 3 shows the CEA classification. Fig. 3 shows a controlled environment agriculture classification analysis.

Through their capacity to maintain ideal temperatures in a variety of climatic conditions, these resources and technologies significantly contribute to the improvement of energy efficiency, environmental sustainability, and quality of life.

### 1.3 Some challenges and considerations regarding temperature control

Some challenges to controlling temperature in the environment are given by.

1. It might be difficult to monitor energy use and maintain effective temperature regulation. Energy-intensive industrial cooling procedures and HVAC systems can have a negative influence on the environment and lead to expensive operating expenses. Adopting energy-efficient technology is critical, and examples include building automation, smart HVAC systems, and passive design techniques. By modifying heating and cooling according to actual demands, these strategies optimize energy usage and lower overall energy consumption and greenhouse gas emissions.
2. Climate change complicates temperature management by altering weather patterns and increasing the frequency of intense heat waves and volatile temperature swings. Moreover, the Infrastructure and natural systems must adapt to these changes to maintain operating performance and protect ecosystems. Developing strong technology-cluster strategies (TCS) requires integrating adaptive technology, bolstering emergency preparedness, and prolonging infrastructure lifespan to mitigate climate-related dangers and maintain environmental stability.
3. For many people and organizations, the initial expenses of installing and maintaining TCSs especially those with energy-efficient technologies can be unaffordable. A major obstacle still exists in affordability, especially in emerging or low-income areas with constrained financial resources. Promoting financial incentives, subsidies, and financing choices for energy-efficient devices is one way to address cost issues. The widespread use and sustainability of these technologies may be promoted by government efforts, collaborations with financial institutions, and community-based programmed that assist lower the cost and increase accessibility.

Some other challenges are the urban heat island effect and technology advancement and adaptation.

### 1.4 Possible solutions to the above-discussed challenges

A comprehensive strategy that incorporates energy efficiency, climate resilience, environmental stewardship, and community participation is needed to address the complex issues of temperature regulation in the environment. Using cutting-edge HVAC technology, such as smart systems and energy recovery ventilation, in conjunction with building retrofits and tenant education on energy-saving techniques, is crucial to addressing excessive energy use and promoting efficiency. In addition to promoting cool roof technologies and reflecting pavements, boosting green infrastructure such as urban forests and green roofs is one way to mitigate the urban heat island effect. Creating robust infrastructure with heat-resistant materials and incorporating adaptive technology into water management and HVAC systems are essential for adapting to the effects of climate change. Refrigerant-related environmental effects can be reduced by switching to low-global warming potential substitutes and putting closed-loop cooling systems in place. Water-efficient technology and community-based water stewardship activities should be given priority in water resource management plans. To ensure successful implementation and operation, technological improvements should priorities the research and development of sustainable solutions, bolstered by training programmed. To remove financial obstacles and make sustainable technology affordable, creative funding strategies and financial incentives are needed. Proactive involvement and strong monitoring methods should be used to guarantee compliance with regulatory criteria. To encourage behavior, change and the adoption of sustainable practices, public awareness campaigns and educational programmed are essential. The integration of varied viewpoints and skills is facilitated by interdisciplinary collaboration among stakeholders, which promotes innovation and sustainable growth in temperature control methods.

## 2 Literature review

In this section of the manuscript, we discuss the literature review in different steps.

### 2.1 Literature review of temperature control system and application

An examination of the literature on TCS demonstrates the wide range of approaches and technology available for improving indoor climate control. Numerous studies have examined traditional HVAC systems, emphasizing their excessive energy usage and negative environmental effects. However, recent studies have concentrated more on environmentally friendly substitutes like geothermal heating and cooling, which achieve great efficiency by utilizing the steady temperatures of the soil. The potential of passive solar architecture to use building orientation and materials to naturally control temperatures has been studied, to minimize the need for mechanical systems. The comfort and indoor air quality of radiant floor heating and natural ventilation systems have been well recognized. Research on evaporative cooling systems and green roofs shows that they can reduce energy consumption in dry conditions and lessen urban heat islands. Many researchers gave their ideas on the application, solutions and challenges of TCS. Over time the invention of TCS increases and becomes a global issue right now. So, the contributions of many researchers show the significance of TCS globally. All ideas related to TCS have owned significance. We discussed here some of the most popular and significant ideas on TCS analysis and techniques. Some related ideas and theories are discussed below. Khalid and Omatu [1] introduce the concept of a neural network controller to control the TCS. Similarly,

Mantovani and Ferrarini [2] discussed an assessment of temperature control of commercial buildings with model predictive control techniques. Kondoh et al. [3] describe the development of TCS for liquid droplets using surface acoustic wave devices. Liu et al. [4] introduced green data centers with IoT sensing and cloud-assisted smart TCS. Soyguder and Alli [5] gave significance related to an expert system for the humidity and TC in HVAC systems using ANFIS and optimization. Moreover, the idea of infrared TCS for a completely noncontact polymerase chain reaction in microfluidic chips was given by Roper et al. [6]. Zhang et al. [7] discussed the concept of the development of temperature and humidity-independent control (THIC) air-conditioning systems in China. Model-based temperature controller development for water-cooled PEM fuel cell systems was discussed by Saygili et al. [8]. Miralles et al. [9] gave an overview of heating and TC in microfluidic systems. Hidayat et al. [10] proposed the new concept of TCS and the design of temperature monitoring and control systems in smart hen coop based on the Internet of Things.

### 2.2 Literature review of multi-attribute group decision-making in fuzzy environments

Fuzzy set theory (FST) developed by Zadeh [11] has great significance in different fields of life because of its advance mathematical structure and framework. Many researchers utilize FST in different fields of life and they give their ideas in different fields. Keeping in mind the importance of fuzzy structure, Dequan et al. [12] introduce the concept of application of expert fuzzy PID method for temperature control of the heating furnace. Pacco [13] introduced a simulation of TC and irrigation time in the production of tulips using fuzzy logic. Moreover, Purwanto et al. [14] created the design of server room temperature and humidity control systems using fuzzy logic based on microcontrollers. The idea to design a room temperature and humidity controller using fuzzy logic was given by Das et al. [15]. Kannan et al. [16] delivered the linear diophantine fuzzy CODAS method to describe the importance of fuzzy structure in MADM problems. Al-shami et al. [17] described the SR-Fuzzy sets and their AOs to propose their application in fuzzy decision-making. No doubt FST has great significance but due to its limitations to discuss only membership degree (MD), the structure of intuitionistic fuzzy set (IFS) [18] was developed by Atanassov. IFS uses the MD as well as non-membership degree (ND) to cover the limitation of FST. Numerous scholars utilize IFSs in different fields. Xu and Yager [19] introduced some geometric AOs based on IFSs. Senapati et al. [20] developed Aczel-Alsina AOs based on IFS and proposed its applications in MADM. Xu and Wang [21] induced generalized AOs for IFSs and their application in GDM. Wang and Liu [22] initiated IF geometric AOs based on Einstein operations. Yu and Xu [23] discussed prioritized IF AOs. Huang [24] gave more work and discussed the concept of IF Hamacher AOs and their application to MADM. Imran et al. [25] proposed the MADM approach for robot selection based on interval-valued IFS. The idea of IFS was limited due to its limited condition because whenever decision-makers decided to take the value 0.5 for MD and 0.6 for ND then (0.5+0.6∉[0,1]). To make it possible the notion of the Pythagorean fuzzy set (PyFS) [26] was developed by Yager that contains the condition that $\left(MD^{2}+ND^{2}\in \left[0,1\right]\right)$. Similar FST and IFS, the idea of PyFS has great applications in different fields. Keeping in mind the advanced conditions of PyFSs and the idea of an improved fuzzy multi-criteria algorithm for optimizing concentrated solar power hybridized systems based on PyFSs given by Xu [27]. Garg [28] discussed the analysis of new logarithmic operational laws, their AOs and their applications for PyFSs. Khan et al. [29] introduced the PyF Dombi AOs and their application in the decision support system. Gul et al. [30] established an extended VIKOR approach based on bipolar fuzzy structure. Also, some algebraic structures based on T-bipolar soft set have been developed by Mahmood et al. [31]. Due to the limitation of PyFS noticed by Yager, the idea of a more generalized structure of a q-rung orthopair fuzzy set (q-ROFS) [32] has been developed. The idea of q-ROFS is the more generalized form in FST. Some of the applications are discussed here. Garg and Chen [33] discussed MAGDM based on neutrality AOs of q-ROFSs. Based on Yager t-norm and t-conorm, Akram and Shahzadi [34] produced the notion of Yager AOs for q-ROFNs. A robust q-ROFS information AOs using Einstein operations with application to sustainable energy planning decision management was discussed by Riaz et al. [35]. Moreover based on q-ROFSs, Seikh and Mandal [36] proposed Archimedean AOs and used these notions in the site selection for software operating units. Moreover, Seikh and Mandal [37] also discussed the q-ROF Frank AOs and utilized these ideas to MADM with unknown weights. Seikh and Mandal [38] proposed a MAGDM approach for quasirung orthopair fuzzy sets. Naz et al. [39] developed an integrated CRITIC MABAC method for 2-tuple linguistic cubic q-ROFSs and discussed its applications to MADM problems. Also, Naz et al. [40] explored the MADM approach to study astronomy in the probabilistic linguistic q-ROF VOKOR technique. Naz et al. [41] utilized the neural language processing technique and utilized this method for product selection. Naz et al. [42] discussed the CILOS-WASPAS approach based on Schweizer-Sklar power AOs for evaluating cosmetic brands.

The theory of complex fuzzy set (CFS) is another extension of fuzzy set first invented by Ramot et al. [43] in 2022 in polar form and then presented by Tamir et al. [44] in Cartesian form. Bi et al. [45,46] discussed the idea of CF geometric and arithmetic AOs. To discuss the MD as well as ND in the Cartesian form of complex numbers the notion of the complex intuitionistic fuzzy set (CIFS) has been developed by Ali et al. [47]. Garg and Rani [48] utilized the notion of CIFs and discussed average and geometric AOs and proposed its MCDM approach. Rani and Garg [49] introduced the idea of CIF power AOs for solving MCDM problems. The polar and Cartesian forms of complex Pythagorean fuzzy sets (CPyFSs) have been developed in Refs. [50,51]. Based on these ideas, the idea of CPy Dombi fuzzy AOs and their DM was developed by Akram et al. [52]. Moreover, Akram et al. [53] introduce the prioritized weighted AOs under CPyF information.

## 3 Motivation

The notion of the Cartesian form of CPyFS introduced by Ullah et al. [50] contradicts the basic concept of the crisp set theory (CST) and fuzzy set theory (FST) because, in CST and FST, an element fully belongs to a set if its membership value is 1 and does not belong to set if its membership value is zero. In this regard, Ullah et al. [50] structure is less applicable and non-reliable. According to the basic concept, if an element fully belongs to a set then its membership value must be 1+ι1 and its non-membership value must be 0+ι0, and in this case $\sqrt{1+1}=1.414⩽̸1$ and the basic condition of Ullah et al. [50] structure is violated. It means that Ullah et al. [50] theory contradicts the basic concept of belonging and non-belonging. Moreover whenever we discuss the notion of the Cartesian form of CPyFS introduced by Labassi et al. [51], then this notion is limited because whenever the decision-makers treat the data in the form of (0.9+ι0.7,0.8+ι0.7), then the basic condition developed by the Labassi et al. [51] is violated. So based on these observations, both of these structures are non-reliable or limited in either case. In this case, there is a need to define such a powerful notion that can handle the limitation and non-reliability of both structures.

To critically evaluate the limitations of the polar form of CPyFS for practical applications such as temperature control systems, we can focus on several areas.

1. For effective temperature adjustment and maintenance, temperature control systems frequently need fast reaction times and simple interpretability. Particularly in real-time applications, the employment of magnitude and angle in the polar form in CPyFS makes interpretation more difficult. Converting actual temperature readings into angular components, or phases, could cause needless delay and complication.
2. For example, using polar coordinates to get the ideal temperature setting is less apparent than using the Cartesian form, because membership degrees are directly related to the system's particular variables.
3. Temperature fluctuations in real-world systems require exact and tiny adjustments, frequently within fractions of a degree, particularly in delicate applications like semiconductor fabrication or chemical reactors. Because polar forms employ trigonometric transformations to indicate uncertainties, they may induce approximation mistakes. This may result in fewer accurate corrections and inadequate temperature regulation.
4. For simplicity and efficiency, digital controllers that mostly rely on Cartesian representations are the foundation of many control systems, including temperature regulators. Computational overhead is increased when CPyFS is implemented in polar form because it frequently requires back-and-forth conversions between polar and Cartesian coordinates. The system's overall efficiency may be lowered by this extra computational burden.

### 3.1 The literature gap of existing notions

1. If we discuss the notion of a complex intuitionistic fuzzy set [47] in Cartesian form, this notion is limited in terms of its structure. No doubt the proposed structure in Ref. [47] can discuss the MD and ND but it can never discuss the data like (0.3+ι0.7,0.9+ι0.4) because notice that (0.3+0.9∉[0,1]and0.7+0.4∉[0,1]). To address this issue, we have to define a structure that can cover all such issues because there is a chance of data lost in existing notions.
2 The notion produced by Labassi et al. [51] is nevertheless the Cartesian form and it can cover the limitation that exists in the notion of complex intuitionistic fuzzy set introduced by Ali et al. [47]. But if we discuss the data given in the set {(0.6+ι0.9,0.8+ι0.7)}, then we can see that the basic condition for the notion produced by Labassi et al. [51] is $\left(0.9^{2}+0.7^{2}=1.3\notin \left[0,1\right]\right)$ can never work for this kind of data. Hence in this regard, we think of producing such kind of structure that can cover that kind of data loss and more advanced information can be covered.
3. There is a structure that can only discuss the MD in Cartesian form like the notion of Tamir et al. [44], but the ND is ignored in this regard. In many situations, we have to discuss the MD as well as ND in one structure to discuss both viewpoints. In this regard, there is a need to define such a structure that can cover such kind of advanced data and both kinds of aspects like MD and NMD can be covered in one structure.
4. When we replace $\prime \prime q^{\prime \prime }$ with 1 and 2 then developed Cq-ROFYWA and Cq-ROFYWG AOs reduce to Cartesian forms of CIFYWA, CIFYWG, CPyFYWA, and CPyFYWG AOs.

### 3.2 Section-wise study

In section 1, we have developed an introduction to the proposed theory. Section 2 is developed to define a literature review of the existing theory and section 3 is about the motivation of the introduced theory. In section 4, we have discussed the preliminaries that can help to define the further theory. In section 5, we have introduced a novel idea of the Cartesian form of Cq-ROFS. Section 6 is deduced to define the Yager operational laws based on Cq-ROFSs and aggregation theory. In section 7, we have developed the MAGDM technique along with illustrative analysis. Section 8 is about the comparative analysis and sensitivity analysis of the introduced theory. In section 9, we have discussed the conclusion.

## 4 Preliminaries

In this section, we have investigated the idea of the Cartesian form of CFS, q-ROFS and Yager operational laws for q-ROFNs.

Tamir et al. [44] produced the Cartesian form of CFS and utilized the complex unit square instead of the complex unit disc. The main idea is presented in the definition given below.

Definition 1 [44]: Let U be the universal. Then the notion of the Cartesian form of CFS is given by

CFS={(x:(Q(x)))|x∈U}

={(x:(Q(x)=A(x)+ιB(x)))|x∈U}

Where Q(x)=A(x)+ιB(x) is called MD.

The notion of q-ROFS was introduced by Yager [32] which can generalize the theory of IFS and PyFS.

Definition 2 [32]: The notion of q-ROFS is defined by

q−ROFS={(x:(Q(x),R(x)))|x∈U}

Where Q:U→[0,1] and R:U→[0,1] is the MD and ND respectively with condition $0\leq Q{\left(x\right)}^{q}+R{\left(x\right)}^{q}\leq 1$. For simplicity q−ROFS={Q(x),R(x)} is called q-ROF number.

Based on Yager t-norm and t-conorm, Akram and Shahzadi [34] produced the basic operations called Yager operational laws for q-ROFNs that can further help in defining the aggregation theory. Akram and Shahzadi [34] introduced the q-ROF Yager weighted average and geometric AOs. These valuable notions are given by.

Definition 3 [34]: Let ${q-ROF}_{1}=\left\{\left(A_{1},C_{1}\right)\right\}$ and ${q-ROF}_{2}=\left\{\left(A_{2},C_{2}\right)\right\}$ be two Cq-ROFNs and γ>0,p>0 and q>0. Then based on q-ROFNS, Yager's operations are defined by

1. ${q-ROF}_{1}⨁{q-ROF}_{2}=\left\{\begin{array}{c} \left(\sqrt[{q}]{\min\left(1,{\left({\left(A_{1}\right)}^{q\gamma }+{\left(A_{2}\right)}^{q\gamma }\right)}^{\frac{1}{\gamma }}\right)}\right)\text{,}\\ \left(\sqrt[{q}]{1-\min\left(1,{\left({\left(1-{\left(C_{1}\right)}^{q}\right)}^{\gamma }+{\left(1-{\left(C_{2}\right)}^{q}\right)}^{\gamma }\right)}^{\frac{1}{\gamma }}\right)}\right) \end{array}\right\}$
2. ${q-ROF}_{1}⨂{q-ROF}_{2}==\left\{\left(\begin{array}{c} \sqrt[{q}]{1-\min\left(1,{\left({\left(1-{\left(A_{1}\right)}^{q}\right)}^{\gamma }+{\left(1-{\left(A_{2}\right)}^{q}\right)}^{\gamma }\right)}^{\frac{1}{\gamma }}\right)},\\ \sqrt[{q}]{\min\left(1,{\left({\left(C_{1}\right)}^{q\gamma }+{\left(C_{2}\right)}^{q\gamma }\right)}^{\frac{1}{\gamma }}\right)} \end{array}\right)\right\}$
3. $p{q-ROF}_{1}=\left\{\left(\sqrt[{q}]{\min\left(1,{\left({\left(pA_{1}\right)}^{q\gamma }\right)}^{\frac{1}{\gamma }}\right)},\sqrt[{q}]{1-\min\left(1,{\left(p{\left(1-{\left(C_{1}\right)}^{q}\right)}^{\gamma }\right)}^{\frac{1}{\gamma }}\right)}\right)\right\}$
4. ${\left({q-ROF}_{1}\right)}^{p}=\left\{\left(\sqrt[{q}]{1-\min\left(1,{\left(p{\left(1-{\left(A_{1}\right)}^{q}\right)}^{\gamma }\right)}^{\frac{1}{\gamma }}\right)},\sqrt[{q}]{\min\left(1,{\left({\left(pC_{1}\right)}^{q\gamma }\right)}^{\frac{1}{\gamma }}\right)}\right)\right\}$

## 5 Novel complex q-rung orthopair fuzzy set

The notion of the Cartesian form of CPyFS introduced by Ullah et al. [43] contradicts the basic concept of the CST and FST because in CST and FST, an element fully belongs to a set if its membership value is 1 and does not belong to set if its membership value is zero. In this regard, Ullah et al. [50] structure is less applicable and non-reliable. According to the basic concept, if an element fully belongs to a set then its membership value must be 1+ι1, and its non-membership value must be 0+ι0 and in this case $\sqrt{1+1}=1.414⩽̸1$ and the basic condition of Ullah et al. [50] structure is violated. It means that Ullah et al. [50] theory contradicts the basic concept of belonging and non-belonging. Moreover, whenever we discuss the notion of the Cartesian form of CPyFS introduced by Labassi et al. [51], then this notion is limited because whenever the decision-makers take the data in the form of (0.9+ι0.7,0.8+ι0.7), then the basic condition developed by the Labassi et al. [51] is violated.

So based on these observations, here we have to develop the idea of the Cartesian form of Cq-ROFS.

Definition 4 Let U be the universal set. Then the notion of Cq-ROFS in Cartesian form is given by

Cq−ROF={x:Q(x),R(x)|x∈U}

Where Q(x) is the membership degree (MD) and R(x) is a non-membership degree (ND) located in the complex unit square. Note that Q(x)=A(x)+ιB(x) with A(x),B(x)∈[0,1] and R(x)=C(x)+ιD(x) with C(x),D(x)∈[0,1]. Then def. 4 is called Cq-ROFS under the condition that $0\leq A{\left(x\right)}^{q}+C{\left(x\right)}^{q}\leq 1$ and $0\leq B{\left(x\right)}^{q}+D{\left(x\right)}^{q}\leq 1$ for q≥1. For the sake of simplicity, Cq−ROF={(A+ιB,C+ιD)} is called complex q-rung orthopair fuzzy number (Cq-ROFN) and in our further discussion we will utilize this notation for our convenience.

Remark 1 Note that the proposed notion is more generalized because

1. When we use q=1 then the initiated notion is reduced to the structure presented in Ref. [40].
2. Similarly, when we utilize q=2, then the introduced notions degenerate into CPyFS in the Cartesian form given in Ref. [44].

Definition 5 Let ${Cq-ROF}_{1}=\left\{\left(A_{1}+\iota B_{1},C_{1}+\iota D_{1}\right)\right\}$ be the notion of Cartesian form of Cq-ROFN. Then the idea of score and accuracy function is defined by

$$Sr.\left(Cq-ROF_{1}\right)=\frac{1}{4}\left(2+{\left(A_{1}\right)}^{q}+{\left(B_{1}\right)}^{q}-{\left(C_{1}\right)}^{q}-{\left(D_{1}\right)}^{q}\right)\;Where\;Sr.\left(Cq-ROF_{1}\right)\in \left[0,1\right]$$

Also.

$Ac.\left(Cq-ROF_{1}\right)=\frac{1}{4}\left(2+{\left(A_{1}\right)}^{q}+{\left(B_{1}\right)}^{q}+{\left(C_{1}\right)}^{q}+{\left(D_{1}\right)}^{q}\right)$ Where $Ac.\left(Cq-ROF_{1}\right)\in \left[0,1\right]$

## 6 Complex q-rung orthopair fuzzy Yager aggregation operators

Definition 6 Let ${Cq-ROF}_{1}=\left\{\left(A_{1}+\iota B_{1},C_{1}+\iota D_{1}\right)\right\}$ and ${Cq-ROF}_{2}=\left\{\left(A_{2}+\iota B_{2},C_{2}+\iota D_{2}\right)\right\}$ be two Cq-ROFNs and γ>0,p>0 and q>0. The Yager operations are defined as follows.

1. ${Cq-ROF}_{1}⨁{Cq-ROF}_{2}=\left\{\left(\begin{array}{c} \sqrt[{q}]{\min\left(1,{\left({\left(A_{1}\right)}^{q\gamma }+{\left(A_{2}\right)}^{q\gamma }\right)}^{\frac{1}{\gamma }}\right)}+\iota \sqrt[{q}]{\min\left(1,{\left({\left(B_{1}\right)}^{q\gamma }+{\left(B_{2}\right)}^{q\gamma }\right)}^{\frac{1}{\gamma }}\right)},\\ \sqrt[{q}]{1-\min\left(1,{\left({\left(1-{\left(C_{1}\right)}^{q}\right)}^{\gamma }+{\left(1-{\left(C_{2}\right)}^{q}\right)}^{\gamma }\right)}^{\frac{1}{\gamma }}\right)}+\\ \iota \sqrt[{q}]{1-\min\left(1,{\left({\left(1-{\left(D_{1}\right)}^{q}\right)}^{\gamma }+{\left(1-{\left(D_{2}\right)}^{q}\right)}^{\gamma }\right)}^{\frac{1}{\gamma }}\right)} \end{array}\right)\right\}$
2. ${Cq-ROF}_{1}⨂{Cq-ROF}_{2}==\left\{\left(\begin{array}{c} \left(\begin{array}{c} \sqrt[{q}]{1-\min\left(1,{\left({\left(1-{\left(A_{1}\right)}^{q}\right)}^{\gamma }+{\left(1-{\left(A_{2}\right)}^{q}\right)}^{\gamma }\right)}^{\frac{1}{\gamma }}\right)}+\\ \iota \sqrt[{q}]{1-\min\left(1,{\left({\left(1-{\left(B_{1}\right)}^{q}\right)}^{\gamma }+{\left(1-{\left(B_{2}\right)}^{q}\right)}^{\gamma }\right)}^{\frac{1}{\gamma }}\right)} \end{array}\right),\\ \left(\sqrt[{q}]{\min\left(1,{\left({\left(C_{1}\right)}^{q\gamma }+{\left(C_{2}\right)}^{q\gamma }\right)}^{\frac{1}{\gamma }}\right)}+\iota \sqrt[{q}]{\min\left(1,{\left({\left(D_{1}\right)}^{q\gamma }+{\left(D_{2}\right)}^{q\gamma }\right)}^{\frac{1}{\gamma }}\right)}\right) \end{array}\right)\right\}$
3. $p{Cq-ROF}_{1}=\left\{\left(\begin{array}{c} \sqrt[{q}]{\min\left(1,{\left({\left(pA_{1}\right)}^{q\gamma }\right)}^{\frac{1}{\gamma }}\right)}+\iota \sqrt[{q}]{\min\left(1,{\left({\left(pB_{1}\right)}^{q\gamma }\right)}^{\frac{1}{\gamma }}\right)},\\ \sqrt[{q}]{1-\min\left(1,{\left(p{\left(1-{\left(C_{1}\right)}^{q}\right)}^{\gamma }\right)}^{\frac{1}{\gamma }}\right)}+\\ \iota \sqrt[{q}]{1-\min\left(1,{\left(p{\left(1-{\left(D_{1}\right)}^{q}\right)}^{\gamma }\right)}^{\frac{1}{\gamma }}\right)} \end{array}\right)\right\}\text{,}$
4. ${\left({Cq-ROF}_{1}\right)}^{p}=\left\{\left(\begin{array}{c} \left(\sqrt[{q}]{1-\min\left(1,{\left(p{\left(1-{\left(A_{1}\right)}^{q}\right)}^{\gamma }\right)}^{\frac{1}{\gamma }}\right)}+\iota \sqrt[{q}]{1-\min\left(1,{\left(p{\left(1-{\left(B_{1}\right)}^{q}\right)}^{\gamma }\right)}^{\frac{1}{\gamma }}\right)}\right),\\ \left(\sqrt[{q}]{\min\left(1,{\left({\left(pC_{1}\right)}^{q\gamma }\right)}^{\frac{1}{\gamma }}\right)}+\iota \sqrt[{q}]{\min\left(1,{\left({\left(pD_{1}\right)}^{q\gamma }\right)}^{\frac{1}{\gamma }}\right)}\right) \end{array}\right)\right\}$

Now utilizing the operational laws given in def. 6, we can define AOs under the environment of Cq-ROFNs as follows.

Definition 7 Let ${Cq-ROF}_{i}=\left\{\left(A_{i}+\iota B_{i},C_{i}+\iota D_{i}\right)\right\}\;\left(i=1,2,\ldots ,m\right)$ be the collection of Cq-ROFNs. Then the notion of the Cq-ROFYWA operator is defined by a mapping $M:R^{m}\to R$ such that

$$Cq-ROFYWA\left({Cq-ROF}_{1},{Cq-ROF}_{2},{Cq-ROF}_{3},\ldots ,{Cq-ROF}_{m}\right)={⊕}_{i=1}^{m}\left(\omega_{i}{Cq-ROF}_{i}\right)$$

Where $\omega ={\left(\omega_{1},\omega_{q},\omega_{3},\ldots ,\omega_{m}\right)}^{Τ}$ is the weight vector (WV) of Cq−ROFNs and $\sum_{i=1}^{m}\omega_{i}=1\text{.}$

Theorem 1 Assume that ${\text{Cq}-\text{ROF}}_{i}=\left\{\left(A_{i}+\iota B_{i},C_{i}+\iota D_{i}\right)\right\}\;\left(i=1,2,\ldots ,m\right)$ denotes the family of Cq-ROFNs. The overall result obtained through the Cq-ROFYWA operator is again Cq-ROFN and $\text{Cq}-\text{ROFYWA}\left({\text{Cq}-\text{ROF}}_{1},{\text{Cq}-\text{ROF}}_{2},{\text{Cq}-\text{ROF}}_{3},\ldots ,{\text{Cq}-\text{ROF}}_{m}\right)={⊕}_{i=1}^{m}\left(\omega_{i}{\text{Cq}-\text{ROF}}_{i}\right)$

(1) $$=\left\{\left(\begin{array}{c} \sqrt[{q}]{\min\left(1,{\left(\sum_{i=1}^{m}\left({\omega_{i}\left(A_{i}\right)}^{q\gamma }\right)\right)}^{\frac{1}{\gamma }}\right)}+\iota \sqrt[{q}]{\min\left(1,{\left(\sum_{i=1}^{m}\left({\omega_{i}\left(B_{i}\right)}^{q\gamma }\right)\right)}^{\frac{1}{\gamma }}\right)},\\ \sqrt[{q}]{1-\min\left(1,{\left(\sum_{i=1}^{m}\left(\omega_{i}{\left(1-{\left(C_{i}\right)}^{q}\right)}^{\gamma }\right)\right)}^{\frac{1}{\gamma }}\right)}+\iota \sqrt[{q}]{1-\min\left(1,{\left(\sum_{i=1}^{m}\left(\omega_{i}{\left(1-{\left(D_{i}\right)}^{q}\right)}^{\gamma }\right)\right)}^{\frac{1}{\gamma }}\right)} \end{array}\right)\right\}$$

Proof: To prove this result we will use the mathematical induction technique.

**Step 1:** For m=2,
  $$\omega_{1}{CPyFR}_{1}=\left\{\left(\begin{array}{c} \sqrt[{q}]{\min\left(1,{\left({\omega_{1}\left(A_{1}\right)}^{q\gamma }\right)}^{\frac{1}{\gamma }}\right)}+\iota \sqrt[{q}]{\min\left(1,{\left({\omega_{1}\left(B_{1}\right)}^{q\gamma }\right)}^{\frac{1}{\gamma }}\right)},\\ \sqrt[{q}]{1-\min\left(1,{\left(\omega_{1}{\left(1-{\left(C_{1}\right)}^{q}\right)}^{\gamma }\right)}^{\frac{1}{\gamma }}\right)}+\iota \sqrt[{q}]{1-\min\left(1,{\left(\omega_{1}{\left(1-{\left(D_{1}\right)}^{q}\right)}^{\gamma }\right)}^{\frac{1}{\gamma }}\right)} \end{array}\right)\right\}\;and$$
  $$\omega_{2}{Cq-ROF}_{2}=\left\{\left(\begin{array}{c} \sqrt[{q}]{\min\left(1,{\left({\omega_{2}\left(A_{2}\right)}^{q\gamma }\right)}^{\frac{1}{\gamma }}\right)}+\iota \sqrt[{q}]{\min\left(1,{\left({\omega_{2}\left(B_{2}\right)}^{q\gamma }\right)}^{\frac{1}{\gamma }}\right)},\\ \sqrt[{q}]{1-\min\left(1,{\left(\omega_{2}{\left(1-{\left(C_{2}\right)}^{q}\right)}^{\gamma }\right)}^{\frac{1}{\gamma }}\right)}+\iota \sqrt[{q}]{1-\min\left(1,{\left(\omega_{2}{\left(1-{\left(D_{2}\right)}^{q}\right)}^{\gamma }\right)}^{\frac{1}{\gamma }}\right)} \end{array}\right)\right\}$$

Therefore, we get

$$\omega_{1}{Cq-ROF}_{1}⨁\omega_{2}{Cq-ROF}_{2}=\left\{\left(\begin{array}{c} \sqrt[{q}]{\min\left(1,{\left({\omega_{1}\left(A_{1}\right)}^{q\gamma }\right)}^{\frac{1}{\gamma }}\right)}+\iota \sqrt[{q}]{\min\left(1,{\left({\omega_{1}\left(B_{1}\right)}^{q\gamma }\right)}^{\frac{1}{\gamma }}\right)},\\ \sqrt[{q}]{1-\min\left(1,{\left(\omega_{1}{\left(1-{\left(C_{1}\right)}^{q}\right)}^{\gamma }\right)}^{\frac{1}{\gamma }}\right)}+\iota \sqrt[{q}]{1-\min\left(1,{\left(\omega_{1}{\left(1-{\left(D_{1}\right)}^{q}\right)}^{\gamma }\right)}^{\frac{1}{\gamma }}\right)} \end{array}\right)\right\}$$

$$⨁\left\{\left(\begin{array}{c} \sqrt[{q}]{\min\left(1,{\left({\omega_{2}\left(A_{2}\right)}^{q\gamma }\right)}^{\frac{1}{\gamma }}\right)}+\iota \sqrt[{q}]{\min\left(1,{\left({\omega_{2}\left(B_{2}\right)}^{q\gamma }\right)}^{\frac{1}{\gamma }}\right)},\\ \sqrt[{q}]{1-\min\left(1,{\left(\omega_{2}{\left(1-{\left(C_{2}\right)}^{q}\right)}^{\gamma }\right)}^{\frac{1}{\gamma }}\right)}+\iota \sqrt[{q}]{1-\min\left(1,{\left(\omega_{2}{\left(1-{\left(D_{2}\right)}^{q}\right)}^{\gamma }\right)}^{\frac{1}{\gamma }}\right)} \end{array}\right)\right\}$$

$$=\left\{\begin{array}{c} \left(\begin{array}{c} \sqrt[{q}]{\min\left(1,{\left(\omega_{1}{\left(A_{1}\right)}^{q\gamma }+\omega_{q}{\left(A_{2}\right)}^{q\gamma }\right)}^{\frac{1}{\gamma }}\right)}+\\ \iota \sqrt[{q}]{\min\left(1,{\left(\omega_{1}{\left(B_{1}\right)}^{q\gamma }+\omega_{q}{\left(B_{2}\right)}^{q\gamma }\right)}^{\frac{1}{\gamma }}\right)},\\ \sqrt[{q}]{1-\min\left(1,{\left({\omega_{1}\left(1-{\left(C_{1}\right)}^{q}\right)}^{\gamma }+\omega_{q}{\left(1-{\left(C_{2}\right)}^{q}\right)}^{\gamma }\right)}^{\frac{1}{\gamma }}\right)}+\\ \iota \sqrt[{q}]{1-\min\left(1,{\left({\omega_{1}\left(1-{\left(D_{1}\right)}^{q}\right)}^{\gamma }+\omega_{q}{\left(1-{\left(D_{2}\right)}^{q}\right)}^{\gamma }\right)}^{\frac{1}{\gamma }}\right)} \end{array}\right)\text{,} \end{array}\right\}$$

$$=\left\{\left(\begin{array}{c} \sqrt[{q}]{\min\left(1,{\left(\sum_{i=1}^{2}\left({\omega_{i}\left(A_{i}\right)}^{q\gamma }\right)\right)}^{\frac{1}{\gamma }}\right)}+\iota \sqrt[{q}]{\min\left(1,{\left(\sum_{i=1}^{2}\left({\omega_{i}\left(B_{i}\right)}^{q\gamma }\right)\right)}^{\frac{1}{\gamma }}\right)},\\ \sqrt[{q}]{1-\min\left(1,{\left(\sum_{i=1}^{2}\left(\omega_{i}{\left(1-{\left(C_{i}\right)}^{q}\right)}^{\gamma }\right)\right)}^{\frac{1}{\gamma }}\right)}+\iota \sqrt[{q}]{1-\min\left(1,{\left(\sum_{i=1}^{2}\left(\omega_{i}{\left(1-{\left(D_{i}\right)}^{q}\right)}^{\gamma }\right)\right)}^{\frac{1}{\gamma }}\right)} \end{array}\right)\right\}$$

**Step 2:** Assume that eq. (1) is valid for m=k that is

$$Cq-ROFYWA\left({Cq-ROF}_{1},Cq-ROF_{2},Cq-ROF_{3},\ldots ,{Cq-ROF}_{m}\right)=\left\{\left(\begin{array}{c} \sqrt[{q}]{\min\left(1,{\left(\sum_{i=1}^{k}\left({\omega_{i}\left(A_{i}\right)}^{q\gamma }\right)\right)}^{\frac{1}{\gamma }}\right)}+\iota \sqrt[{q}]{\min\left(1,{\left(\sum_{i=1}^{k}\left({\omega_{i}\left(B_{i}\right)}^{q\gamma }\right)\right)}^{\frac{1}{\gamma }}\right)},\\ \sqrt[{q}]{1-\min\left(1,{\left(\sum_{i=1}^{k}\left(\omega_{i}{\left(1-{\left(C_{i}\right)}^{q}\right)}^{\gamma }\right)\right)}^{\frac{1}{\gamma }}\right)}+\iota \sqrt[{q}]{1-\min\left(1,{\left(\sum_{i=1}^{k}\left(\omega_{i}{\left(1-{\left(D_{i}\right)}^{q}\right)}^{\gamma }\right)\right)}^{\frac{1}{\gamma }}\right)} \end{array}\right)\right\}$$

Now for m=k+1, we get

$$Cq-ROFYWA\left({Cq-ROF}_{1},Cq-ROF_{2},Cq-ROF_{3},\ldots ,{Cq-ROF}_{m}\right)=\left\{\left(\begin{array}{c} \sqrt[{q}]{\min\left(1,{\left(\sum_{i=1}^{k}\left({\omega_{i}\left(A_{i}\right)}^{q\gamma }\right)\right)}^{\frac{1}{\gamma }}\right)}+\iota \sqrt[{q}]{\min\left(1,{\left(\sum_{i=1}^{k}\left({\omega_{i}\left(B_{i}\right)}^{q\gamma }\right)\right)}^{\frac{1}{\gamma }}\right)},\\ \sqrt[{q}]{1-\min\left(1,{\left(\sum_{i=1}^{k}\left(\omega_{i}{\left(1-{\left(C_{i}\right)}^{q}\right)}^{\gamma }\right)\right)}^{\frac{1}{\gamma }}\right)}+\iota \sqrt[{q}]{1-\min\left(1,{\left(\sum_{i=1}^{k}\left(\omega_{i}{\left(1-{\left(D_{i}\right)}^{q}\right)}^{q\gamma }\right)\right)}^{\frac{1}{\gamma }}\right)} \end{array}\right)\right\}$$

$$⨁\left\{\left(\begin{array}{c} \sqrt[{q}]{\min\left(1,{\left({\omega_{k+1}\left(A_{k+1}\right)}^{q\gamma }\right)}^{\frac{1}{\gamma }}\right)}+\iota \sqrt[{q}]{\min\left(1,{\left({\omega_{k+1}\left(B_{k+1}\right)}^{q\gamma }\right)}^{\frac{1}{\gamma }}\right)},\\ \sqrt[{q}]{1-\min\left(1,{\left(\omega_{k+1}{\left(1-{\left(C_{k+1}\right)}^{q}\right)}^{\gamma }\right)}^{\frac{1}{\gamma }}\right)}+\iota \sqrt[{q}]{1-\min\left(1,{\left(\omega_{k+1}{\left(1-{\left(D_{k+1}\right)}^{q}\right)}^{\gamma }\right)}^{\frac{1}{\gamma }}\right)} \end{array}\right)\right\}$$

$$=\left\{\left(\begin{array}{c} \sqrt[{q}]{\min\left(1,{\left(\sum_{i=1}^{k+1}\left({\omega_{i}\left(A_{i}\right)}^{q\gamma }\right)\right)}^{\frac{1}{\gamma }}\right)}+\iota \sqrt[{q}]{\min\left(1,{\left(\sum_{i=1}^{k+1}\left({\omega_{i}\left(B_{i}\right)}^{q\gamma }\right)\right)}^{\frac{1}{\gamma }}\right)},\\ \sqrt[{q}]{1-\min\left(1,{\left(\sum_{i=1}^{k+1}\left(\omega_{i}{\left(1-{\left(C_{i}\right)}^{q}\right)}^{\gamma }\right)\right)}^{\frac{1}{\gamma }}\right)}+\iota \sqrt[{q}]{1-\min\left(1,{\left(\sum_{i=1}^{k+1}\left(\omega_{i}{\left(1-{\left(D_{i}\right)}^{q}\right)}^{\gamma }\right)\right)}^{\frac{1}{\gamma }}\right)} \end{array}\right)\right\}$$

Hence eq. (1) is true for m=k+1. So the result is valid for all m.

Next, we have to discuss the basic properties of the proposed theory. The theories are given as follows.

Theorem 2 **(Idempotency)** If all Cq-ROFNs are the same that is ${\text{Cq}-\text{ROF}}_{i}=\left\{\left(A_{i}+\iota B_{i},C_{i}+\iota D_{i}\right)\right\}=\text{Cq}-\text{ROF}=\left\{\left(A+\iota B,C+\iota D\right)\right\}$ then

$$Cq-ROFYWA\left({Cq-ROF}_{1},{Cq-ROF}_{2},{Cq-ROF}_{3},\ldots ,{Cq-ROF}_{m}\right)=Cq-ROF$$

Proof: As ${Cq-ROF}_{i}=\left\{\left(A_{i}+\iota B_{i},C_{i}+\iota D_{i}\right)\right\}=Cq-ROF=\left\{\left(A+\iota B,C+\iota D\right)\right\}$. Then by utilizing eq. (1), we get $Cq-ROFYWA\left({Cq-ROF}_{1},{Cq-ROF}_{2},{Cq-ROF}_{3},\ldots ,{Cq-ROF}_{m}\right)={⊕}_{i=1}^{m}\left(\omega_{i}{Cq-ROF}_{i}\right)$

$$=\left\{\left(\begin{array}{c} \sqrt[{q}]{\min\left(1,{\left(\sum_{i=1}^{m}\left({\omega_{i}\left(A_{i}\right)}^{q\gamma }\right)\right)}^{\frac{1}{\gamma }}\right)}+\iota \sqrt[{q}]{\min\left(1,{\left(\sum_{i=1}^{m}\left({\omega_{i}\left(B_{i}\right)}^{q\gamma }\right)\right)}^{\frac{1}{\gamma }}\right)},\\ \sqrt[{q}]{1-\min\left(1,{\left(\sum_{i=1}^{m}\left(\omega_{i}{\left(1-{\left(C_{i}\right)}^{q}\right)}^{\gamma }\right)\right)}^{\frac{1}{\gamma }}\right)}+\iota \sqrt[{q}]{1-\min\left(1,{\left(\sum_{i=1}^{m}\left(\omega_{i}{\left(1-{\left(D_{i}\right)}^{q}\right)}^{\gamma }\right)\right)}^{\frac{1}{\gamma }}\right)} \end{array}\right)\right\}$$

$$=\left\{\left(\begin{array}{c} \sqrt[{q}]{\min\left(1,{\left({\left(A\right)}^{q\gamma }\right)}^{\frac{1}{\gamma }}\right)}+\iota \sqrt[{q}]{\min\left(1,{\left({\left(B\right)}^{q\gamma }\right)}^{\frac{1}{\gamma }}\right)},\\ \sqrt[{q}]{1-\min\left(1,{\left({\left(1-{\left(C\right)}^{q}\right)}^{\gamma }\right)}^{\frac{1}{\gamma }}\right)}+\iota \sqrt[{q}]{1-\min\left(1,{\left({\left(1-{\left(D\right)}^{q}\right)}^{\gamma }\right)}^{\frac{1}{\gamma }}\right)} \end{array}\right)\right\}$$

$$=\left\{\left(\begin{array}{c} \sqrt[{q}]{\min\left(1,{\left(A\right)}^{q}\right)}+\iota \sqrt[{q}]{\min\left(1,{\left(B\right)}^{q}\right)},\\ \sqrt[{q}]{1-\min\left(1,\left(1-{\left(C\right)}^{q}\right)\right)}+\iota \sqrt[{q}]{1-\min\left(1,\left(1-{\left(D\right)}^{q}\right)\right)} \end{array}\right)\right\}$$

$$=\left\{\left(\begin{array}{c} \sqrt[{q}]{{\left(A\right)}^{q}}+\iota \sqrt[{q}]{{\left(B\right)}^{q}},\\ \sqrt[{q}]{1-\left(1-{\left(C\right)}^{q}\right)}+\iota \sqrt[{q}]{1-\left(1-{\left(D\right)}^{q}\right)} \end{array}\right)\right\}$$

$$=\left\{\left(\begin{array}{c} \left(A\right)+\iota \left(B\right),\\ \left(C\right)+\iota \left(D\right) \end{array}\right)\right\}=Cq-ROF$$

Theorem 3 **(Monotonicity)** Let ${\text{Cq}-\text{ROF}}_{i}=\left\{\left(A_{i}+\iota B_{i},C_{i}+\iota D_{i}\right)\right\},{\text{Cq}-\text{ROF}}_{i}^{*}=\left\{\left(A_{i}^{*}+\iota B_{i}^{*},C_{i}^{*}+\iota D_{i}^{*}\right)\right\}$ be two families of Cq-ROFNs. If $A_{i}^{*}\leq A_{i},B_{i}^{*}\leq B_{i}$ and $C_{i}^{*}\geq C_{i},D_{i}^{*}\geq D_{i}$ for all i, then

$$Cq-ROFYWA\left({Cq-ROF}_{1}^{*},{Cq-ROF}_{2}^{*},{Cq-ROF}_{3}^{*},\ldots ,{Cq-ROF}_{m}^{*}\right)\leq Cq-ROFYWA\left({Cq-ROF}_{1},{Cq-ROF}_{2},{Cq-ROF}_{3},\ldots ,{Cq-ROF}_{m}\right)$$

Proof: First of all we prove this for the real part of MD. Now as $A_{i}^{*}\leq A_{i}$ then

$${\left(\sum_{i=1}^{m}\left({\omega_{i}\left(A_{i}^{*}\right)}^{q\gamma }\right)\right)}^{\frac{1}{\gamma }}\leq {\left(\sum_{i=1}^{m}\left({\omega_{i}\left(A_{i}^{*}\right)}^{q\gamma }\right)\right)}^{\frac{1}{\gamma }}$$

$$\min\left(1,{\left(\sum_{i=1}^{m}\left({\omega_{i}\left(A_{i}^{*}\right)}^{q\gamma }\right)\right)}^{\frac{1}{\gamma }}\right)\leq \min\left(1,{\left(\sum_{i=1}^{m}\left({\omega_{i}\left(A_{i}^{*}\right)}^{q\gamma }\right)\right)}^{\frac{1}{\gamma }}\right)$$

$$\sqrt[{q}]{\min\left(1,{\left(\sum_{i=1}^{m}\left({\omega_{i}\left(A_{i}^{*}\right)}^{q\gamma }\right)\right)}^{\frac{1}{\gamma }}\right)}\leq \sqrt[{q}]{\min\left(1,{\left(\sum_{i=1}^{m}\left({\omega_{i}\left(A_{i}^{*}\right)}^{q\gamma }\right)\right)}^{\frac{1}{\gamma }}\right)}$$

Also as $B_{i}^{*}\leq B_{i}$ then

$${\left(\sum_{i=1}^{m}\left({\omega_{i}\left(B_{i}^{*}\right)}^{q\gamma }\right)\right)}^{\frac{1}{\gamma }}\leq {\left(\sum_{i=1}^{m}\left({\omega_{i}\left(B_{i}^{*}\right)}^{q\gamma }\right)\right)}^{\frac{1}{\gamma }}$$

$$\min\left(1,{\left(\sum_{i=1}^{m}\left({\omega_{i}\left(B_{i}^{*}\right)}^{q\gamma }\right)\right)}^{\frac{1}{\gamma }}\right)\leq \min\left(1,{\left(\sum_{i=1}^{m}\left({\omega_{i}\left(B_{i}^{*}\right)}^{q\gamma }\right)\right)}^{\frac{1}{\gamma }}\right)$$

$$\sqrt[{q}]{\min\left(1,{\left(\sum_{i=1}^{m}\left({\omega_{i}\left(B_{i}^{*}\right)}^{q\gamma }\right)\right)}^{\frac{1}{\gamma }}\right)}\leq \sqrt[{q}]{\min\left(1,{\left(\sum_{i=1}^{m}\left({\omega_{i}\left(B_{i}^{*}\right)}^{q\gamma }\right)\right)}^{\frac{1}{\gamma }}\right)}$$

Similarly, we can prove that

$$\sqrt[{q}]{1-\min\left(1,{\left(\sum_{i=1}^{m}\left(\omega_{i}{\left(1-{\left(C_{i}^{*}\right)}^{q}\right)}^{\gamma }\right)\right)}^{\frac{1}{\gamma }}\right)}\geq \sqrt[{q}]{1-\min\left(1,{\left(\sum_{i=1}^{m}\left(\omega_{i}{\left(1-{\left(C_{i}\right)}^{q}\right)}^{\gamma }\right)\right)}^{\frac{1}{\gamma }}\right)}$$

And

$$\sqrt[{q}]{1-\min\left(1,{\left(\sum_{i=1}^{m}\left(\omega_{i}{\left(1-{\left(D_{i}^{*}\right)}^{q}\right)}^{\gamma }\right)\right)}^{\frac{1}{\gamma }}\right)}\geq \sqrt[{q}]{1-\min\left(1,{\left(\sum_{i=1}^{m}\left(\omega_{i}{\left(1-{\left(D_{i}\right)}^{q}\right)}^{\gamma }\right)\right)}^{\frac{1}{\gamma }}\right)}$$

Hence

$$Cq-ROFYWA\left({Cq-ROF}_{1}^{*},{Cq-ROF}_{2}^{*},{Cq-ROF}_{3}^{*},\ldots ,{Cq-ROF}_{m}^{*}\right)\leq Cq-ROFYWA\left({Cq-ROF}_{1},{Cq-ROF}_{2},{Cq-ROF}_{3},\ldots ,{Cq-ROF}_{m}\right)$$

Theorem 4 **(Boundedness)**: Let ${\text{Cq}-\text{ROF}}_{i}=\left\{\left(A_{i}+\iota B_{i},C_{i}+\iota D_{i}\right)\right\},{\text{Cq}-\text{ROF}}_{i}^{*}=\left\{\left(A_{i}^{*}+\iota B_{i}^{*},C_{i}^{*}+\iota D_{i}^{*}\right)\right\}$ be two families of Cq-ROFNs. Let ${\text{Cq}-\text{ROF}}_{i}^{-}=\min\left({\text{Cq}-\text{ROF}}_{1},{\text{Cq}-\text{ROF}}_{2},\ldots ,{\text{Cq}-\text{ROF}}_{m}\right)$ and ${\text{Cq}-\text{ROF}}_{i}^{+}=\max\left({\text{Cq}-\text{ROF}}_{1},{\text{Cq}-\text{ROF}}_{2},\ldots ,{\text{Cq}-\text{ROF}}_{m}\right)$ where ${\text{Cq}-\text{ROF}}_{i}^{-}=\min\left({\text{Cq}-\text{ROF}}_{1},{\text{Cq}-\text{ROF}}_{2},\ldots ,{\text{Cq}-\text{ROF}}_{m}\right)=\left\{\left(\min\left(A_{i}\right)+\iota \;\min\left(B_{i}\right),\max\left(C_{i}\right)+\iota \;\max\left(D_{i}\right)\right)\right\}$ and ${\text{Cq}-\text{ROF}}_{i}^{+}=\max\left({\text{Cq}-\text{ROF}}_{1},{\text{Cq}-\text{ROF}}_{2},\ldots ,{\text{Cq}-\text{ROF}}_{m}\right)=\left\{\left(\max\left(A_{i}\right)+\iota \;\max\left(B_{i}\right),\min\left(C_{i}\right)+\iota \;\min\left(D_{i}\right)\right)\right\}\text{.}$ Then

$${Cq-ROF}_{i}^{-}\leq Cq-ROFYWA\left({Cq-ROF}_{1},{Cq-ROF}_{2},{Cq-ROF}_{3},\ldots ,{Cq-ROF}_{m}\right)\leq {Cq-ROF}_{i}^{+}\text{.}$$

Proof: This theorem can be proved by using utilizing theorem (2) and theorem (3).

Now we define the notion of Cq-ROFY ordered weighted average (Cq-ROFYOWA) AOs. The prosed theory is given by.

Definition 8 Let ${Cq-ROF}_{i}=\left\{\left(A_{i}+\iota B_{i},C_{i}+\iota D_{i}\right)\right\}\;\left(i=1,2,\ldots ,m\right)$ be the collection of Cq-ROFNs. Then the notion of the Cq-ROFYOWA operator is defined by a mapping $M:R^{m}\to R$ such that

$$Cq-ROFYOWA\left({Cq-ROF}_{1},{Cq-ROF}_{2},{Cq-ROF}_{3},\ldots ,{Cq-ROF}_{m}\right)={⊕}_{i=1}^{m}\left(\omega_{i}{Cq-ROF}_{\vartheta \left(i\right)}\right)$$

Where $\omega ={\left(\omega_{1},\omega_{q},\omega_{3},\ldots ,\omega_{m}\right)}^{Τ}$ is the WV of Cq−ROFNs and $\sum_{i=1}^{m}\omega_{i}=1$ and (ϑ(1),ϑ(2),ϑ(3),…,ϑ(m)) is the permutation of (i=1,2,3,..,m) such that ${Cq-ROF}_{\vartheta \left(i-1\right)}\geq {Cq-ROF}_{\vartheta \left(i\right)}$ for all i=1,2,…,m.

Theorem 5 Assume that ${\text{Cq}-\text{ROF}}_{i}=\left\{\left(A_{i}+\iota B_{i},C_{i}+\iota D_{i}\right)\right\}\;\left(i=1,2,\ldots ,m\right)$ denotes the family of Cq-ROFNs. The overall result obtained through the Cq-ROFYOWA operator is again Cq-ROFN and $\text{Cq}-\text{ROFYOWA}\left({\text{Cq}-\text{ROF}}_{1},{\text{Cq}-\text{ROF}}_{2},{\text{Cq}-\text{ROF}}_{3},\ldots ,{\text{Cq}-\text{ROF}}_{m}\right)={⊕}_{i=1}^{m}\left(\omega_{i}{\text{Cq}-\text{ROF}}_{\vartheta \left(i\right)}\right)$

(2) $$=\left\{\left(\begin{array}{c} \sqrt[{q}]{\min\left(1,{\left(\sum_{i=1}^{m}\left({\omega_{i}\left(A_{\vartheta \left(i\right)}\right)}^{q\gamma }\right)\right)}^{\frac{1}{\gamma }}\right)}+\iota \sqrt[{q}]{\min\left(1,{\left(\sum_{i=1}^{m}\left({\omega_{i}\left(B_{\vartheta \left(i\right)}\right)}^{q\gamma }\right)\right)}^{\frac{1}{\gamma }}\right)},\\ \sqrt[{q}]{1-\min\left(1,{\left(\sum_{i=1}^{m}\left(\omega_{i}{\left(1-{\left(C_{\vartheta \left(i\right)}\right)}^{q}\right)}^{\gamma }\right)\right)}^{\frac{1}{\gamma }}\right)}+\iota \sqrt[{q}]{1-\min\left(1,{\left(\sum_{i=1}^{m}\left(\omega_{i}{\left(1-{\left(D_{\vartheta \left(i\right)}\right)}^{q}\right)}^{\gamma }\right)\right)}^{\frac{1}{\gamma }}\right)} \end{array}\right)\right\}$$

Theorem 6 A Cq-ROFYHWA operator is defined by a mapping $M:R^{m}\to R$ with WVs $\omega ={\left(\omega_{1},\omega_{q},\omega_{3},\ldots ,\omega_{m}\right)}^{Τ}$ and $\sum_{i=1}^{m}\omega_{i}=1$ such that $Cq-ROFYHWA\left({Cq-ROF}_{1},{Cq-ROF}_{2},{Cq-ROF}_{3},\ldots ,{Cq-ROF}_{m}\right)={⊕}_{i=1}^{m}\left(\omega_{i}{Cq-ROF}_{\vartheta \left(i\right)}^{*}\right)$

(3) $$=\left\{\left(\begin{array}{c} \sqrt[{q}]{\min\left(1,{\left(\sum_{i=1}^{m}\left({\omega_{i}\left(A_{\vartheta \left(i\right)}^{*}\right)}^{q\gamma }\right)\right)}^{\frac{1}{\gamma }}\right)}+\iota \sqrt[{q}]{\min\left(1,{\left(\sum_{i=1}^{m}\left({\omega_{i}\left(B_{\vartheta \left(i\right)}^{*}\right)}^{q\gamma }\right)\right)}^{\frac{1}{\gamma }}\right)},\\ \sqrt[{q}]{1-\min\left(1,{\left(\sum_{i=1}^{m}\left(\omega_{i}{\left(1-{\left(C_{\vartheta \left(i\right)}^{*}\right)}^{q}\right)}^{\gamma }\right)\right)}^{\frac{1}{\gamma }}\right)}+\iota \sqrt[{q}]{1-\min\left(1,{\left(\sum_{i=1}^{m}\left(\omega_{i}{\left(1-{\left(D_{\vartheta \left(i\right)}^{*}\right)}^{q}\right)}^{\gamma }\right)\right)}^{\frac{1}{\gamma }}\right)} \end{array}\right)\right\}$$

Where ${\text{Cq}-\text{ROF}}_{\vartheta \left(i\right)}^{*}$ is ith largest weighted Cq-ROFN ${\text{Cq}-\text{ROF}}_{i}^{*}\left({\text{Cq}-\text{ROF}}_{i}^{*}=m\omega_{i}\text{Cq}-\text{RO}F_{i};i=1,2,..,m\right)$ and m is a balancing coefficient.

Remark 2 By using $\omega ={\left(\frac{1}{m},\frac{1}{m},\ldots ,\frac{1}{m}\right)}^{T}$ then Cq−ROFYHWA AOs degenerate into Cq−ROFYWAandCq−ROFYOWA AOs. Thus we can observe that Cq−ROFYHWA AOs are the generalization of Cq−ROFYWAandCq−ROFYOWA AOs.

Definition 9 Let ${Cq-ROF}_{i}=\left\{\left(A_{i}+\iota B_{i},C_{i}+\iota D_{i}\right)\right\}\;\left(i=1,2,\ldots ,m\right)$ be the collection of Cq-ROFNs. Then the notion of the Cq-ROFYWG operator is defined by a mapping $M:R^{m}\to R$ such that

$$Cq-ROFYWG\left({Cq-ROF}_{1},{Cq-ROF}_{2},{Cq-ROF}_{3},\ldots ,{Cq-ROF}_{m}\right)={⨂}_{i=1}^{m}\left({Cq-ROF}_{i}^{\omega_{i}}\right)$$

Where $\omega ={\left(\omega_{1},\omega_{q},\omega_{3},\ldots ,\omega_{m}\right)}^{Τ}$ is the WVs of Cq−ROFNs and $\sum_{i=1}^{m}\omega_{i}=1\text{.}$

Theorem 7 Let ${\text{Cq}-\text{ROF}}_{i}=\left\{\left(A_{i}+\iota B_{i},C_{i}+\iota D_{i}\right)\right\}\;\left(i=1,2,\ldots ,m\right)$ be the collection of Cq-ROFNs. The outcome obtained through the Cq-ROFYWG operator is again Cq-ROFN $Cq-ROFYWG\left({Cq-ROF}_{1},{Cq-ROF}_{2},{Cq-ROF}_{3},\ldots ,{Cq-ROF}_{m}\right)={⨂}_{i=1}^{m}\left({{Cq-ROF}_{i}}^{\omega_{i}}\right)$

$$=\left\{\left(\begin{array}{c} \sqrt[{q}]{1-\min\left(1,{\left(\sum_{i=1}^{m}\left(\omega_{i}{\left(1-{\left(A_{i}\right)}^{q}\right)}^{\gamma }\right)\right)}^{\frac{1}{\gamma }}\right)}+\iota \sqrt[{q}]{1-\min\left(1,{\left(\sum_{i=1}^{m}\left(\omega_{i}{\left(1-{\left(B_{i}\right)}^{q}\right)}^{\gamma }\right)\right)}^{\frac{1}{\gamma }}\right)},\\ \sqrt[{q}]{\min\left(1,{\left(\sum_{i=1}^{m}\left({\omega_{i}\left(C_{i}\right)}^{q\gamma }\right)\right)}^{\frac{1}{\gamma }}\right)}+\iota \sqrt[{q}]{\min\left(1,{\left(\sum_{i=1}^{m}\left({\omega_{i}\left(D_{i}\right)}^{q\gamma }\right)\right)}^{\frac{1}{\gamma }}\right)} \end{array}\right)\right\}$$

Proof: Same as Theorem (2).

Definition 10 Let ${Cq-ROF}_{i}=\left\{\left(A_{i}+\iota B_{i},C_{i}+\iota D_{i}\right)\right\}\;\left(i=1,2,\ldots ,m\right)$ be the collection of Cq-ROFNs. Then the notion of the Cq-ROFYOWG operator is defined by a mapping $M:R^{m}\to R$ such that

$$Cq-ROFYOWG\left({Cq-ROF}_{1},{Cq-ROF}_{2},{Cq-ROF}_{3},\ldots ,{Cq-ROF}_{m}\right)={⨂}_{i=1}^{m}\left({Cq-ROF}_{\vartheta \left(i\right)}^{\omega_{i}}\right)$$

Where $\omega ={\left(\omega_{1},\omega_{q},\omega_{3},\ldots ,\omega_{m}\right)}^{Τ}$ is the WV of Cq−ROFNs and $\sum_{i=1}^{m}\omega_{i}=1$ and (ϑ(1),ϑ(2),ϑ(3),…,ϑ(m)) is the permutation of (i=1,2,3,..,m) such that ${Cq-ROF}_{\vartheta \left(i-1\right)}\geq {Cq-ROF}_{\vartheta \left(i\right)}$ for all i=1,2,…,m.

Theorem 8 Let ${\text{Cq}-\text{ROF}}_{i}=\left\{\left(A_{i}+\iota B_{i},C_{i}+\iota D_{i}\right)\right\}\;\left(i=1,2,\ldots ,m\right)$ be the collection of Cq-ROFNs. The outcome obtained through the Cq-ROFYOWG operator is again Cq-ROFN $\text{Cq}-\text{ROFYOWG}\left({\text{Cq}-\text{ROF}}_{1},{\text{Cq}-\text{ROF}}_{2},{\text{Cq}-\text{ROF}}_{3},\ldots ,{\text{Cq}-\text{ROF}}_{m}\right)={⨂}_{i=1}^{m}\left({{\text{Cq}-\text{ROF}}_{\vartheta \left(i\right)}}^{\omega_{i}}\right)$

$$=\left\{\left(\begin{array}{c} \sqrt[{q}]{1-\min\left(1,{\left(\sum_{i=1}^{m}\left(\omega_{i}{\left(1-{\left(A_{\vartheta \left(i\right)}\right)}^{q}\right)}^{\gamma }\right)\right)}^{\frac{1}{\gamma }}\right)}+\iota \sqrt[{q}]{1-\min\left(1,{\left(\sum_{i=1}^{m}\left(\omega_{i}{\left(1-{\left(B_{\vartheta \left(i\right)}\right)}^{q}\right)}^{\gamma }\right)\right)}^{\frac{1}{\gamma }}\right)},\\ \sqrt[{q}]{\min\left(1,{\left(\sum_{i=1}^{m}\left({\omega_{i}\left(C_{\vartheta \left(i\right)}\right)}^{q\gamma }\right)\right)}^{\frac{1}{\gamma }}\right)}+\iota \sqrt[{q}]{\min\left(1,{\left(\sum_{i=1}^{m}\left({\omega_{i}\left(D_{\vartheta \left(i\right)}\right)}^{q\gamma }\right)\right)}^{\frac{1}{\gamma }}\right)} \end{array}\right)\right\}$$

Theorem 9 A Cq-ROFYHWG operator is defined by a mapping $M:R^{m}\to R$ with WVs $\omega ={\left(\omega_{1},\omega_{q},\omega_{3},\ldots ,\omega_{m}\right)}^{Τ}$ and $\sum_{i=1}^{m}\omega_{i}=1$ such that $\text{Cq}-\text{ROFYHWG}\left({\text{Cq}-\text{ROF}}_{1},{\text{Cq}-\text{ROF}}_{2},{\text{Cq}-\text{ROF}}_{3},\ldots ,{\text{Cq}-\text{ROF}}_{m}\right)={⨂}_{i=1}^{m}\left({{\text{Cq}-\text{ROF}}_{\vartheta \left(i\right)}^{*}}^{\omega_{i}}\right)$

$$=\left\{\left(\begin{array}{c} \sqrt[{q}]{1-\min\left(1,{\left(\sum_{i=1}^{m}\left(\omega_{i}{\left(1-{\left(A_{\vartheta \left(i\right)}^{*}\right)}^{q}\right)}^{\gamma }\right)\right)}^{\frac{1}{\gamma }}\right)}+\iota \sqrt[{q}]{1-\min\left(1,{\left(\sum_{i=1}^{m}\left(\omega_{i}{\left(1-{\left(B_{\vartheta \left(i\right)}^{*}\right)}^{q}\right)}^{\gamma }\right)\right)}^{\frac{1}{\gamma }}\right)},\\ \sqrt[{q}]{\min\left(1,{\left(\sum_{i=1}^{m}\left({\omega_{i}\left(C_{\vartheta \left(i\right)}^{*}\right)}^{q\gamma }\right)\right)}^{\frac{1}{\gamma }}\right)}+\iota \sqrt[{q}]{\min\left(1,{\left(\sum_{i=1}^{m}\left({\omega_{i}\left(D_{\vartheta \left(i\right)}^{*}\right)}^{q\gamma }\right)\right)}^{\frac{1}{\gamma }}\right)} \end{array}\right)\right\}$$

Where ${\text{Cq}-\text{ROF}}_{\vartheta \left(i\right)}^{*}$ is ith largest weighted Cq-ROFN ${\text{Cq}-\text{ROF}}_{i}^{*}\left({\text{Cq}-\text{ROF}}_{i}^{*}=\text{Cq}-\text{RO}F_{i}^{m\omega_{i}};i=1,2,..,m\right)$ and m is a balancing coefficient.

## 7 The MAGDM

Let $\zeta =\left\{\zeta_{1},\zeta_{2},\zeta_{3},\ldots ,\zeta_{n}\right\}$ be the set of n alternatives and $\xi =\left\{\xi_{1},\xi_{2},\ldots ,\xi_{m}\right\}$ denotes m attributes with weight vectors $\omega_{i}\;\left(i=1,2,\ldots ,m\right)\;and\sum_{i=1}^{m}\omega_{i}=1\text{.}$ Let the set of experts be denoted by $\epsilon_{k}=\left\{e_{1},e_{2},\ldots ,e_{r}\right\}$ for k=1,2,…,r with WVs $\left(\varphi_{1},\varphi_{2},\ldots ,\varphi_{r}\right)\;and\sum_{k=1}^{r}\varphi_{r}=1$. Let $M_{j\times i}=\left\{\left(A_{ji}+\iota B_{ji},C_{ji}+\iota D_{ji}\right)\right\}$ be the decision matrix consisting of Cq−ROFNs in Cartesian form. Now for dealing with the MAGDM problem, the overall algorithm is given by

**Step 1:** Assume that decision analyst provide their assessment in the Cartesian form of Cq−ROFNs according to each attribute. So decision matrix is given by
  $$<listaend>M_{j\times i}=\left[\begin{array}{ccc} \left(A_{11}+\iota B_{11},C_{11}+\iota D_{11}\right) & \ldots & \begin{array}{cc} \left(A_{12}+\iota B_{12},C_{12}+\iota D_{12}\right) & \left(A_{1m}+\iota B_{1m},C_{1m}+\iota D_{1m}\right) \end{array}\\ \left(A_{21}+\iota B_{21},C_{21}+\iota D_{21}\right) & \ldots & \begin{array}{cc} \left(A_{22}+\iota B_{22},C_{22}+\iota D_{22}\right) & \left(A_{2m}+\iota B_{2m},C_{2m}+\iota D_{2m}\right) \end{array}\\ \begin{array}{c} \vdots \\ \left(A_{n1}+\iota B_{n1},C_{n1}+\iota D_{n1}\right) \end{array} & \begin{array}{c} \ddots \\ \ldots \end{array} & \begin{array}{cc} \begin{array}{c} \vdots \\ \left(A_{n2}+\iota B_{n2},C_{n2}+\iota D_{n2}\right) \end{array} & \begin{array}{c} \vdots \\ \left(A_{nm}+\iota B_{nm},C_{nm}+\iota D_{nm}\right) \end{array} \end{array} \end{array}\right]$$
**Step 2:** Normalize the decision matrix according to the formula given by
  $$N_{j\times i}=\left\{ \begin{array}{c} \left(A_{ji}+\iota B_{ji},C_{ji}+\iota D_{ji}\right)\;for\;benefit\;type\;\\ \left(C_{ji}+\iota D_{ji},A_{ji}+\iota B_{ji}\right)\;for\;cost\;type\; \end{array}\right.$$

**Step 3:** Find out the combined matrix by utilizing the notion of the Cartesian form of Cq-ROFYWA or Cq-ROFYWG AOs.
**Step 4:** Now aggregate the decision matrix given in Step 3 by using any of the AOs (Cq-ROFYWA or Cq-ROFYWG).
**Step 5:** To decide the best alternative, we have to utilize the formula for score values. So utilize definition (5) for this purpose. Then rank the alternatives for choosing the best alternative.

### 7.1 Illustrative example

In a variety of contexts, such as homes, businesses, and industries, TCSs are critical to preserving comfortable interior environments. Though they have long been the norm, traditional HVAC (heating, ventilation, and air conditioning) systems can have high energy costs and negative environmental effects. Alternative TCSs have attracted attention due to their creative methods and advantages for the environment, particularly as the need for sustainable and energy-efficient solutions increases. These substitutes support international efforts to mitigate climate change by lowering greenhouse gas emissions in addition to energy consumption.

**Fig. 4:**
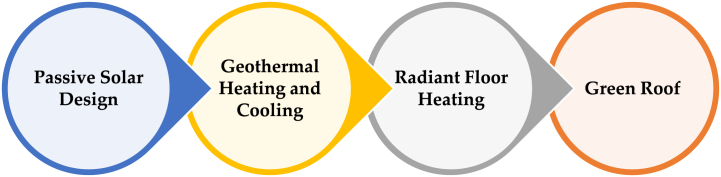
Graphical representation of different Alternatives.

In Fig. 4, there are some notable alternatives to TCS.

Table 1 discusses the different alternatives and their descriptions.

**Table 1:** Different alternatives of TCS.

Notions	Alternatives	Detail Explanation
$\zeta_{1}$	**Passive Solar Design**	Using natural sunshine to control interior temperature is known as passive solar design. Buildings can efficiently absorb, store, and transfer solar energy when windows are positioned and thermal mass materials such as stone or concrete are used. This design uses less energy and has a smaller environmental effect by minimizing the need for mechanical heating and cooling.
$\zeta_{2}$	**Geothermal Heating and Cooling**	The constant temperatures may be used by geothermal systems to heat and cool buildings. These systems use a heat pump and a ground heat exchanger to move heat from the building to the soil. With continuous interior climate management and much lower energy expenditures, geothermal heating and cooling is very efficient.
$\zeta_{3}$	**Radiant Floor Heating**	Radiant floor heating systems involve embedding water pipes or electric heating components under the floor surface. This method evenly circulates heat throughout the room space leading to a cozy and uniform warmth all around. Typically, radiant heating is much more energy efficient than forced air systems that end up blowing warm air to just one part of the room and not even evenly at that hence higher levels of interior comfort can be achieved with less energy consumption.
$\zeta_{4}$	**Green Roofs**	Vegetation-blanketed inexperienced roofs act as herbal insulation and aid in controlling the temperature of buildings. They retain warm temperatures inside the wintry weather and absorb warmness within the summer time to maintain interiors cooler. Green roofs are also useful resources for the surroundings via reducing city heat islands and improving air great.

**Fig. 5:**
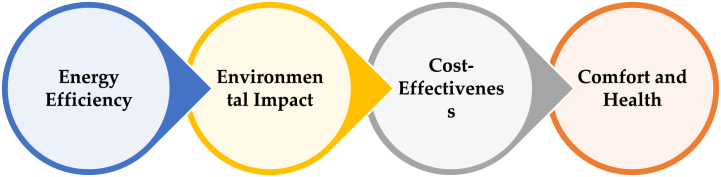
Graphical representation of different Attributes.

Several crucial factors are considered while assessing temperature manipulate systems so that you can ascertain their typical worth, sustainability, and efficacy. These characteristics draw interest to the effectiveness and performance of the structures in addition to their results on environmental and human comfort. By understanding more about those features, we can decide whether systems are extra appropriate for certain software or surroundings. Several vital TCS opportunity characteristics are shown in Fig. 5.

Table 2 describes different attributes.

**Table 2:** Different attributes of TCS.

Notions	Attributes	Detail Explanation
$\xi_{1}$	**Energy Efficiency**	Energy efficiency is a key feature of alternative TCSs. Take geothermal heating and cooling, radiant floor heating, and modern building management systems as examples: these technologies achieve high levels of energy utilization, which significantly slashes consumption. In turn, such solutions curtail wastage and promote sustainable energy practices— all while cutting down on the carbon footprints and utility costs. This is achieved by using natural resources along with cutting-edge technologies that make waste resourceful.
$\xi_{2}$	**Environmental Impact**	TCS's environmental implication is a key issue to consider. In addition to addressing this alternative with green roofs and passive solar architecture, there are several environmental benefits of such developments like evaporative cooling systems. They play a role in reducing the effects of urban heat islands and fostering air quality through greenhouse gas emissions. The other alternatives ensure a healthy living environment and are based on supporting sustainability, which entails using renewable resources that promote natural processes.
$\xi_{3}$	**Cost-Effectiveness**	Another important factor that always have to be focused on in any TCS is its efficiency. Techniques such as radiant floor heating or geothermal heating initially may prove to be very costly, but annually, it is observed that energy and maintenance costs are often replenished by the overall expenses incurred at the initial stage. In addition, energy conservation and utilization measures such as natural ventilation and green roofs may provide tax advantage or any other stimulus for the green building.
$\xi_{4}$	**Comfort and Health**	These are some of the considerations that should be given while evaluating TCSs; comfort and health. Steady heat supply and eradicating drafts are the main benefits that make radiant floor heating enhance the level of comfort of houses. With essential air circulation, natural disclosures increment indoor air quality and decrease the rates of respiratory illnesses and other ailments. Better conditions might be achieved by the incorporation of both PCMs and green roofs to improve internal air quality and thermal comfort.

**Step 1:** Based on the provided attributes, assume that the decision analyst assesses the different alternatives and provides their assessment values in the form of linguistic terms given in Table 3, Table 4, Table 5 where “H” denotes the high, “E” represents exponential, “L” stands for low, “M” for medium and “I” for inadequate. Furthermore, to make these linguistic terms meaningful, the conversion of linguistic terms in the Cartesian form of Cq-ROFNs is given in Table 6.

**Table 3:** Linguistic term provided by expert 1.

	$\xi_{1}$	$\xi_{2}$	$\xi_{3}$	$\xi_{4}$
$\zeta_{1}$	H	I	E	I
$\zeta_{2}$	L	L	H	L
$\zeta_{3}$	M	M	M	M
$\zeta_{4}$	E	H	L	H

**Table 4:** Linguistic term provided by expert 2.

	$\xi_{1}$	$\xi_{2}$	$\xi_{3}$	$\xi_{4}$
$\zeta_{1}$	M	H	H	L
$\zeta_{2}$	E	E	M	M
$\zeta_{3}$	L	L	L	H
$\zeta_{4}$	I	M	E	E

**Table 5:** Linguistic term provided by expert 3.

	$\xi_{1}$	$\xi_{2}$	$\xi_{3}$	$\xi_{4}$
$\zeta_{1}$	I	H	I	I
$\zeta_{2}$	L	M	M	M
$\zeta_{3}$	H	M	H	H
$\zeta_{4}$	M	E	L	E

**Table 6:** Conversion of the linguistic term into the Cartesian form of Cq-ROFNs.

Linguistic terms	Cartesian form of related Cq-ROFNs
H	$\left(\begin{array}{c} 0.6+\iota 0.7\text{,}\\ \;0.5+\iota 0.6 \end{array}\right)$
L	$\left(\begin{array}{c} 0.2+\iota 0.3\text{,}\\ \;0.1+\iota 0.4 \end{array}\right)$
M	$\left(\begin{array}{c} 0.3+\iota 0.4\text{,}\\ \;0.5+\iota 0.6 \end{array}\right)$
E	$\left(\begin{array}{c} 0.6+\iota 0.5\text{,}\\ \;0.2+\iota 0.1 \end{array}\right)$
I	$\left(\begin{array}{c} 0.4+\iota 0.3\text{,}\\ \;0.4+\iota 0.1 \end{array}\right)$

Now the data given by the experts in Table 3, Table 4, Table 5 can be transformed very easily in the shape of the Cartesian form of Cq-RONs so that we can proceed to the next step. Hence Table 7, Table 8, Table 9 shows the transformed form of data given in Table 3, Table 4, Table 5 based on observation of data given in Table 6.

**Table 7:** Transformed form of data given in Table 3 based on Table 6.

	$\xi_{1}$	$\xi_{2}$	$\xi_{3}$	$\xi_{4}$
$\zeta_{1}$	$\left(\begin{array}{c} 0.6+\iota 0.7\text{,}\\ \;0.5+\iota 0.6 \end{array}\right)$	$\left(\begin{array}{c} 0.4+\iota 0.3,\\ 0.4+\iota 0.1 \end{array}\right)$	$\left(\begin{array}{c} 0.6+\iota 0.5\text{,}\\ \;0.2+\iota 0.1 \end{array}\right)$	$\left(\begin{array}{c} 0.4+\iota 0.3\text{,}\\ \;0.4+\iota 0.1 \end{array}\right)$
$\zeta_{2}$	$\left(\begin{array}{c} 0.2+\iota 0.3\text{,}\\ \;0.1+\iota 0.4 \end{array}\right)$	$\left(\begin{array}{c} 0.2+\iota 0.3\text{,}\\ \;0.1+\iota 0.4 \end{array}\right)$	$\left(\begin{array}{c} 0.6+\iota 0.7\text{,}\\ \;0.5+\iota 0.6 \end{array}\right)$	$\left(\begin{array}{c} 0.2+\iota 0.3\text{,}\\ \;0.1+\iota 0.4 \end{array}\right)$
$\zeta_{3}$	$\left(\begin{array}{c} 0.3+\iota 0.4\text{,}\\ \;0.5+\iota 0.6 \end{array}\right)$	$\left(\begin{array}{c} 0.3+\iota 0.4\text{,}\\ \;0.5+\iota 0.6 \end{array}\right)$	$\left(\begin{array}{c} 0.3+\iota 0.4\text{,}\\ \;0.5+\iota 0.6 \end{array}\right)$	$\left(\begin{array}{c} 0.3+\iota 0.4\text{,}\\ \;0.5+\iota 0.6 \end{array}\right)$
$\zeta_{4}$	$\left(\begin{array}{c} 0.6+\iota 0.5\text{,}\\ \;0.2+\iota 0.1 \end{array}\right)$	$\left(\begin{array}{c} 0.6+\iota 0.7\text{,}\\ \;0.5+\iota 0.6 \end{array}\right)$	$\left(\begin{array}{c} 0.2+\iota 0.3\text{,}\\ \;0.1+\iota 0.4 \end{array}\right)$	$\left(\begin{array}{c} 0.6+\iota 0.7\text{,}\\ \;0.5+\iota 0.6 \end{array}\right)$

**Table 8:** Transformed form of data given in Table 4 based on Table 6.

	$\xi_{1}$	$\xi_{2}$	$\xi_{3}$	$\xi_{4}$
$\zeta_{1}$	$\left(\begin{array}{c} 0.3+\iota 0.4\text{,}\\ \;0.5+\iota 0.6 \end{array}\right)$	$\left(\begin{array}{c} 0.6+\iota 0.7\text{,}\\ \;0.5+\iota 0.6 \end{array}\right)$	$\left(\begin{array}{c} 0.6+\iota 0.7\text{,}\\ \;0.5+\iota 0.6 \end{array}\right)$	$\left(\begin{array}{c} 0.2+\iota 0.3\text{,}\\ \;0.1+\iota 0.4 \end{array}\right)$
$\zeta_{2}$	$\left(\begin{array}{c} 0.6+\iota 0.5\text{,}\\ \;0.2+\iota 0.1 \end{array}\right)$	$\left(\begin{array}{c} 0.6+\iota 0.5\text{,}\\ \;0.2+\iota 0.1 \end{array}\right)$	$\left(\begin{array}{c} 0.3+\iota 0.4\text{,}\\ \;0.5+\iota 0.6 \end{array}\right)$	$\left(\begin{array}{c} 0.3+\iota 0.4\text{,}\\ \;0.5+\iota 0.6 \end{array}\right)$
$\zeta_{3}$	$\left(\begin{array}{c} 0.2+\iota 0.3\text{,}\\ \;0.1+\iota 0.4 \end{array}\right)$	$\left(\begin{array}{c} 0.2+\iota 0.3\text{,}\\ \;0.1+\iota 0.4 \end{array}\right)$	$\left(\begin{array}{c} 0.2+\iota 0.3\text{,}\\ \;0.1+\iota 0.4 \end{array}\right)$	$\left(\begin{array}{c} 0.6+\iota 0.7\text{,}\\ \;0.5+\iota 0.6 \end{array}\right)$
$\zeta_{4}$	$\left(\begin{array}{c} 0.4+\iota 0.3\text{,}\\ \;0.4+\iota 0.1 \end{array}\right)$	$\left(\begin{array}{c} 0.3+\iota 0.4\text{,}\\ \;0.5+\iota 0.6 \end{array}\right)$	$\left(\begin{array}{c} 0.6+\iota 0.5\text{,}\\ \;0.2+\iota 0.1 \end{array}\right)$	$\left(\begin{array}{c} 0.6+\iota 0.5\text{,}\\ \;0.2+\iota 0.1 \end{array}\right)$

**Table 9:** Transformed form of data given in Table 5 based on Table 6.

	$\xi_{1}$	$\xi_{2}$	$\xi_{3}$	$\xi_{4}$
$\zeta_{1}$	$\left(\begin{array}{c} 0.4+\iota 0.3\text{,}\\ \;0.4+\iota 0.1 \end{array}\right)$	$\left(\begin{array}{c} 0.6+\iota 0.7\text{,}\\ \;0.5+\iota 0.6 \end{array}\right)$	$\left(\begin{array}{c} 0.4+\iota 0.3\text{,}\\ \;0.4+\iota 0.1 \end{array}\right)$	$\left(\begin{array}{c} 0.4+\iota 0.3\text{,}\\ \;0.4+\iota 0.1 \end{array}\right)$
$\zeta_{2}$	$\left(\begin{array}{c} 0.2+\iota 0.3\text{,}\\ \;0.1+\iota 0.4 \end{array}\right)$	$\left(\begin{array}{c} 0.3+\iota 0.4\text{,}\\ \;0.5+\iota 0.6 \end{array}\right)$	$\left(\begin{array}{c} 0.3+\iota 0.4\text{,}\\ \;0.5+\iota 0.6 \end{array}\right)$	$\left(\begin{array}{c} 0.3+\iota 0.4\text{,}\\ \;0.5+\iota 0.6 \end{array}\right)$
$\zeta_{3}$	$\left(\begin{array}{c} 0.6+\iota 0.7\text{,}\\ \;0.5+\iota 0.6 \end{array}\right)$	$\left(\begin{array}{c} 0.3+\iota 0.4\text{,}\\ \;0.5+\iota 0.6 \end{array}\right)$	$\left(\begin{array}{c} 0.6+\iota 0.7\text{,}\\ \;0.5+\iota 0.6 \end{array}\right)$	$\left(\begin{array}{c} 0.6+\iota 0.7\text{,}\\ \;0.5+\iota 0.6 \end{array}\right)$
$\zeta_{4}$	$\left(\begin{array}{c} 0.3+\iota 0.4\text{,}\\ \;0.5+\iota 0.6 \end{array}\right)$	$\left(\begin{array}{c} 0.6+\iota 0.5\text{,}\\ \;0.2+\iota 0.1 \end{array}\right)$	$\left(\begin{array}{c} 0.2+\iota 0.3\text{,}\\ \;0.1+\iota 0.4 \end{array}\right)$	$\left(\begin{array}{c} 0.6+\iota 0.5\text{,}\\ \;0.2+\iota 0.1 \end{array}\right)$

**Step 2:** Since all attributes are of benefit type so there is no need to normalize the data given in Table 7, Table 8, Table 9

**Step 3:** By using the WV for the experts (0.29,0.39,0.32), combine matrix by using the notion of Cq-ROFYWA AO given in Table 10.

**Table 10:** Combine matrix.

	$\xi_{1}$	$\xi_{2}$	$\xi_{3}$	$\xi_{4}$
$\zeta_{1}$	$\left(\begin{array}{c} 0.3985+\iota 0.5018\text{,}\\ \;0.3226+\iota 0.3591 \end{array}\right)$	$\left(\begin{array}{c} 0.4454+\iota 0.5611\text{,}\\ \;0.3255+\iota 0.3688 \end{array}\right)$	$\left(\begin{array}{c} 0.4430+\iota 0.5214\text{,}\\ \;0.2604+\iota 0.2605 \end{array}\right)$	$\left(\begin{array}{c} 0.2378+\iota 0.1643\text{,}\\ \;0.1947+\iota 0.1553 \end{array}\right)$
$\zeta_{2}$	$\left(\begin{array}{c} 0.4131+\iota 0.3144\text{,}\\ \;0.0609+\iota 0.1947 \end{array}\right)$	$\left(\begin{array}{c} 0.4131+\iota 0.3165\text{,}\\ \;0.1966+\iota 0.2739 \end{array}\right)$	$\left(\begin{array}{c} 0.3981+\iota 0.5018\text{,}\\ \;0.3535+\iota 0.4647 \end{array}\right)$	$\left(\begin{array}{c} 0.1574+\iota 0.2427\text{,}\\ \;0.2892+\iota 0.4037 \end{array}\right)$
$\zeta_{3}$	$\left(\begin{array}{c} 0.4030+\iota 0.5079\text{,}\\ \;0.2657+\iota 0.3832 \end{array}\right)$	$\left(\begin{array}{c} 0.1545+\iota 0.2384\text{,}\\ \;0.2657+\iota 0.3832 \end{array}\right)$	$\left(\begin{array}{c} 0.4030+\iota 0.5079\text{,}\\ \;0.2657+\iota 0.3832 \end{array}\right)$	$\left(\begin{array}{c} 0.4452+\iota 0.5611\text{,}\\ \;0.3535+\iota 0.4647 \end{array}\right)$
$\zeta_{4}$	$\left(\begin{array}{c} 0.3986+\iota 0.3057\text{,}\\ \;0.2528+\iota 0.2340 \end{array}\right)$	$\left(\begin{array}{c} 0.4369+\iota 0.5030\text{,}\\ \;0.2873+\iota 0.3591 \end{array}\right)$	$\left(\begin{array}{c} 0.4131+\iota 0.3144\text{,}\\ \;0.0609+\iota 0.1947 \end{array}\right)$	$\left(\begin{array}{c} 0.4647+\iota 0.5043\text{,}\\ \;0.1941+\iota 0.2221 \end{array}\right)$

**Step 4:** Now utilize the Cartesian form of Cq-ROFYWA or Cq-ROFYWG AO to aggregate the data given in Table 10 with WVs of attributes (0.25,0.21,0.30,0.24). The computed results are given in Table 11.

**Table 11:** Aggregated results by using the Cartesian form of Cq-ROFYWA and Cq-ROFYWG AOs.

By using Cq-ROFYWA AO	By using Cq-ROFYWG AO
$\zeta_{1}=\left(\begin{array}{c} 0.2762+\iota 0.3747,\\ \;0.1500+\iota 0.1666 \end{array}\right)$	$\zeta_{1}=\left(\begin{array}{c} 0.2494+\iota 0.3245,\\ \;0.1683+\iota 0.1994 \end{array}\right)$
$\zeta_{2}=\left(\begin{array}{c} 0.2517+\iota 0.3060,\\ \;0.1422+\iota 0.2230 \end{array}\right)$	$\zeta_{2}=\left(\begin{array}{c} 0.2260+\iota 0.2289,\\ \;0.1824+\iota 0.2772 \end{array}\right)$
$\zeta_{3}=\left(\begin{array}{c} 0.2654+\iota 0.3755,\\ \;0.1570+\iota 0.2574 \end{array}\right)$	$\zeta_{3}=\left(\begin{array}{c} 0.2377+\iota 0.3341,\\ \;0.1780+\iota 0.2742 \end{array}\right)$
$\zeta_{4}=\left(\begin{array}{c} 0.2869+\iota 0.3236,\\ \;0.1035+\iota 0.1319 \end{array}\right)$	$\zeta_{4}=\left(\begin{array}{c} 0.2797+\iota 0.2649,\\ \;0.1305+\iota 0.1773 \end{array}\right)$

**Step 5:** Now utilize the def. (5) to find out the score valued for ranking the alternatives. The score values are given in Table 12.

**Table 12:** Score values for Cq-ROFYWA and Cq-ROFYWG AOs.

Score values for Cq-ROFYWA AO	Ranking order for a Cartesian form of Cq-ROFYWA AO	Score values for Cq-ROFYWG AO	Ranking order for a Cartesian form of Cq-ROFYWG AO
$Sr.\left(\zeta_{1}\right)=0.5164$	$\zeta_{1}\geq \zeta_{4}\geq \zeta_{3}\geq \zeta_{2}$	$Sr.\left(\zeta_{1}\right)=0.5092$	$\zeta_{1}\geq \zeta_{4}\geq \zeta_{3}\geq \zeta_{2}$
$Sr.\left(\zeta_{2}\right)=0.5076$		$Sr.\left(\zeta_{2}\right)=0.4990$	
$Sr.\left(\zeta_{3}\right)=0.5126$		$Sr.\left(\zeta_{3}\right)=0.5061$	
$Sr.\left(\zeta_{4}\right)=0.5135$		$Sr.\left(\zeta_{4}\right)=0.5081$	

## 8 Comparative analysis

This section of the article is devoted to discussing the comparative analysis of the proposed theory with some existing notions to show the authenticity and superiority of the proposed theory. Here we take the data from Table 7, Table 8, Table 9 The overall theory is given by.

Notice that if we discuss the structural benefits of the prosed theory then we can notice that.

1. If we discuss the notion of CIFS [47] in Cartesian form, this notion is limited in terms of its structure. No doubt the proposed structure in Ref. [47] can discuss the MD and ND but it can never discuss the data like (0.3+ι0.7,0.9+ι0.4) because notice that (0.3+0.9∉[0,1]and0.7+0.4∉[0,1]). To address this issue, we have to define such structure that can cover all such issues because there is a chance of data being lost in existing notions. The defined notions can cover all these issues.
2. The notion produced by Labassi et al. [51] is nevertheless the Cartesian form and it can cover the limitation that exists in the notion of CIFS introduced by Ali et al. [47]. But if we discuss the data given in the set {(0.6+ι0.9,0.8+ι0.7)}, we can see that the basic condition for the notion produced by Labassi et al. [51] that is $\left(0.9^{2}+0.7^{2}=1.3\notin \left[0,1\right]\right)$ can never work for this kind of data. Hence in this regard, we think to produce such kind of structure that can cover that kind of data loss and more advanced information can be covered. The developed approach can discuss these issues very easily.
3. There is a structure that can only discuss the MD in Cartesian form like the notion of Tamir [44], but the ND is ignored in this regard. In many situations, we have to discuss the MD as well as ND in one structure to discuss both viewpoints. In this regard, there is a need to define such a structure that can cover such kind of advanced data and both kinds of aspects like MD and NMD can be covered in one structure. The introduced notions can discuss these limitations.
4. The data given in Table 8, Table 9, Table 10, can never be discussed by the notions given in the Tamir et al. [44] method and CIFS proposed in Ref. [47]. Labassi et al. [51] handle this data and the overall results are given in Table 13.

**Table 13:** Overall results.

**Existing and proposed notion**	Score values	Ranking Result
**Ullah et al.** [50] **approach**	No result	No result
**Cartesian form of Complex fuzzy set** [44]	No result	No result
**Cartesian form of Complex intuitionistic fuzzy set** [47]	No result	No result
**Cartesian form of Complex Pythagorean fuzzy Yager weighted average AO**	$Sr.\left(\zeta_{1}\right)=0.5691\text{,}$	$\zeta_{4}\geq \zeta_{1}\geq \zeta_{3}\geq \zeta_{2}$
	$Sr.\left(\zeta_{2}\right)=0.5400\text{,}$	
	$Sr.\left(\zeta_{3}\right)=0.5460\text{,}$	
	$Sr.\left(\zeta_{4}\right)=0.5729$	
**Cartesian form of Complex Pythagorean fuzzy Yager weighted geometric AO**	$Sr.\left(\zeta_{1}\right)=0.5354\text{,}$	$\zeta_{4}\geq \zeta_{1}\geq \zeta_{3}\geq \zeta_{2}$
	$Sr.\left(\zeta_{2}\right)=0.4962\text{,}$	
	$Sr.\left(\zeta_{3}\right)=0.5202\text{,}$	
	$Sr.\left(\zeta_{4}\right)=0.5470$	
**Cartesian form of Complex q-rung orthopair fuzzy Yager weighted average AO (Proposed)**	$Sr.\left(\zeta_{1}\right)=0.5164\text{,}$	$\zeta_{1}\geq \zeta_{4}\geq \zeta_{3}\geq \zeta_{2}$
	$Sr.\left(\zeta_{2}\right)=0.5076\text{,}$	
	$Sr.\left(\zeta_{3}\right)=0.5126\text{,}$	
	$Sr.\left(\zeta_{4}\right)=0.5135$	
**Cartesian form of Complex q-rung orthopair fuzzy Yager weighted geometric AO (Proposed)**	$Sr.\left(\zeta_{1}\right)=0.5092\text{,}$	$\zeta_{1}\geq \zeta_{4}\geq \zeta_{3}\geq \zeta_{2}$
	$Sr.\left(\zeta_{2}\right)=0.4990\text{,}$	
	$Sr.\left(\zeta_{3}\right)=0.5061\text{,}$	
	$Sr.\left(\zeta_{4}\right)=0.5081$
5. The basic idea of belonging and non-belonging says that an element belongs to a set if its membership value is 1 and it does not belong to a set if its membership value is 0. In the case of a complex fuzzy structure, we can say that an element fully belongs to a set if its membership value is 1+ι1 and it does not belong to a set if its membership value is 0+ι0. Now in the case of 1+ι1 we can see that $\sqrt{1+1}=1.414⩽̸1$ and thus the basic condition for the Ullah et al. [50] approach is violated. On the other hand, the developed approach can handle this information easily. Hence we can observe from the above discussion that the initiated approach can provide more space to decision makers and it is dominant to the developed approach.
6. In the case of the Labassi et al. [51] method, we can observe that if the data is given by experts in the form of 0.71+ι0.79 then the Cartesian form of CPyFS fails to handle such kind of information because the basic condition utilized in Labassi et al. [51] approach is violated that is $\sqrt{0.71^{2}+0.79^{2}}==1.3341\notin \left[0,1\right]$. While the developed approach can handle it easily. Hence Initiate work is more reliable and superior.

**Fig. 6:**
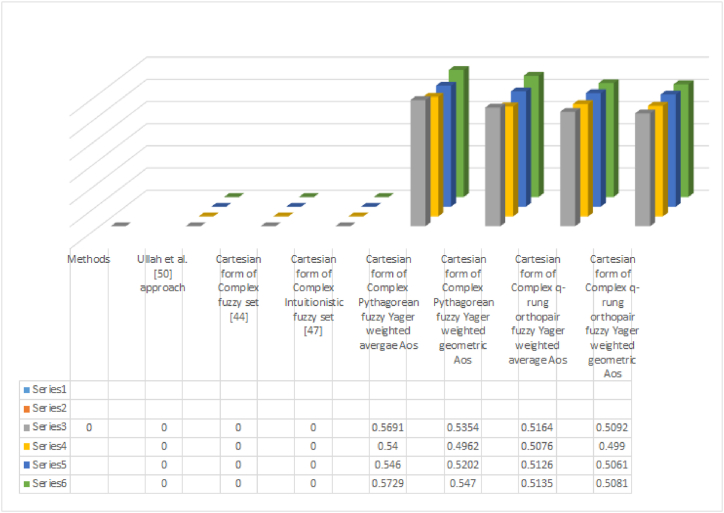
Graphical representation of proposed work given in Table 13.

The graphical analysis of the proposed work is given in Fig. 6.

Moreover, the characteristic analysis of the initiated approach is given in Table 14.

**Table 14:** Characteristic analysis of the delivered approach.

**Methods**	**Considering the second dimension in the data**
Ullah et al. [50] approach	Yes
Cartesian form of Complex fuzzy set [44]	Yes
Cartesian form of Complex intuitionistic fuzzy set [47]	Yes
Cartesian form of Complex Pythagorean fuzzy Yager weighted average AO	Yes
Cartesian form of Complex Pythagorean fuzzy Yager weighted geometric AO	Yes
Cartesian form of Complex q-rung orthopair fuzzy Yager weighted average AO (Proposed)	Yes
Cartesian form of Complex q-rung orthopair fuzzy Yager weighted geometric AO (Proposed)	Yes

### 8.1 Sensitivity analysis

Here we will discuss the effect of parameters on ranking results. The overall results by using different values for the parameter are given in Table 15.

**Table 15:** Sensitivity analysis.

**Parameters values**	**Score values for Cq-ROFYWA AO**	**Ranking result**	**Score values for Cq-ROFYWG AO**	**Ranking result**
For γ=4	$Sr.\left(\zeta_{1}\right)=0.5164\text{,}$	$\zeta_{1}\geq \zeta_{4}\geq \zeta_{3}\geq \zeta_{2}$	$Sr.\left(\zeta_{1}\right)=0.5092\text{,}$	$\zeta_{1}\geq \zeta_{4}\geq \zeta_{3}\geq \zeta_{2}$
	$Sr.\left(\zeta_{2}\right)=0.5076\text{,}$		$Sr.\left(\zeta_{2}\right)=0.4990\text{,}$	
	$Sr.\left(\zeta_{3}\right)=0.5126\text{,}$		$Sr.\left(\zeta_{3}\right)=0.5061\text{,}$	
	$Sr.\left(\zeta_{4}\right)=0.5135\text{,}$		$Sr.\left(\zeta_{4}\right)=0.5081$	
For γ=5	$Sr.\left(\zeta_{1}\right)=0.5170\text{,}$	$\zeta_{1}\geq \zeta_{4}\geq \zeta_{3}\geq \zeta_{2}$	$Sr.\left(\zeta_{1}\right)=0.5087\text{,}$	$\zeta_{1}\geq \zeta_{4}\geq \zeta_{3}\geq \zeta_{2}$
	$Sr.\left(\zeta_{2}\right)=0.5084\text{,}$		$Sr.\left(\zeta_{2}\right)=0.4984\text{,}$	
	$Sr.\left(\zeta_{3}\right)=0.5133\text{,}$		$Sr.\left(\zeta_{3}\right)=0.5054\text{,}$	
	$Sr.\left(\zeta_{4}\right)=0.5141\text{,}$		$Sr.\left(\zeta_{4}\right)=0.5078$	
For γ=6	$Sr.\left(\zeta_{1}\right)=0.5175\text{,}$	$\zeta_{1}\geq \zeta_{4}\geq \zeta_{3}\geq \zeta_{2}$	$Sr.\left(\zeta_{1}\right)=0.5082\text{,}$	$\zeta_{1}\geq \zeta_{4}\geq \zeta_{3}\geq \zeta_{2}$
	$Sr.\left(\zeta_{2}\right)=0.5090\text{,}$		$Sr.\left(\zeta_{2}\right)=0.4979\text{,}$	
	$Sr.\left(\zeta_{3}\right)=0.5138\text{,}$		$Sr.\left(\zeta_{3}\right)=0.5047\text{,}$	
	$Sr.\left(\zeta_{4}\right)=0.5146\text{,}$		$Sr.\left(\zeta_{4}\right)=0.5075$	
For γ=7	$Sr.\left(\zeta_{1}\right)=0.5180\text{,}$	$\zeta_{1}\geq \zeta_{4}\geq \zeta_{3}\geq \zeta_{2}$	$Sr.\left(\zeta_{1}\right)=0.5078\text{,}$	$\zeta_{1}\geq \zeta_{4}\geq \zeta_{3}\geq \zeta_{2}$
	$Sr.\left(\zeta_{2}\right)=0.5095\text{,}$		$Sr.\left(\zeta_{2}\right)=0.4975\text{,}$	
	$Sr.\left(\zeta_{3}\right)=0.5143\text{,}$		$Sr.\left(\zeta_{3}\right)=0.5041\text{,}$	
	$Sr.\left(\zeta_{4}\right)=0.5150\text{,}$		$Sr.\left(\zeta_{4}\right)=0.5072$	
For γ= 8	$Sr.\left(\zeta_{1}\right)=0.5184\text{,}$	$\zeta_{1}\geq \zeta_{4}\geq \zeta_{3}\geq \zeta_{2}$	$Sr.\left(\zeta_{1}\right)=0.5073\text{,}$	$\zeta_{1}\geq \zeta_{4}\geq \zeta_{3}\geq \zeta_{2}$
	$Sr.\left(\zeta_{2}\right)=0.5099\text{,}$		$Sr.\left(\zeta_{2}\right)=0.4971\text{,}$	
	$Sr.\left(\zeta_{3}\right)=0.5147\text{,}$		$Sr.\left(\zeta_{3}\right)=0.5035\text{,}$	
	$Sr.\left(\zeta_{4}\right)=0.5153\text{,}$		$Sr.\left(\zeta_{4}\right)=0.5070$	
For γ=9	$Sr.\left(\zeta_{1}\right)=0.5187\text{,}$	$\zeta_{1}\geq \zeta_{4}\geq \zeta_{3}\geq \zeta_{2}$	$Sr.\left(\zeta_{1}\right)=0.5069\text{,}$	$\zeta_{1}\geq \zeta_{4}\geq \zeta_{3}\geq \zeta_{2}$
	$Sr.\left(\zeta_{2}\right)=0.5102\text{,}$		$Sr.\left(\zeta_{2}\right)=0.4968\text{,}$	
	$Sr.\left(\zeta_{3}\right)=0.5151\text{,}$		$Sr.\left(\zeta_{3}\right)=0.5030\text{,}$	
	$Sr.\left(\zeta_{4}\right)=0.5156\text{,}$		$Sr.\left(\zeta_{4}\right)=0.5068$	
For γ=10	$Sr.\left(\zeta_{1}\right)=0.5190\text{,}$	$\zeta_{1}\geq \zeta_{4}\geq \zeta_{3}\geq \zeta_{2}$	$Sr.\left(\zeta_{1}\right)=0.5065\text{,}$	$\zeta_{1}\geq \zeta_{4}\geq \zeta_{3}\geq \zeta_{2}$
	$Sr.\left(\zeta_{2}\right)=0.5105\text{,}$		$Sr.\left(\zeta_{2}\right)=0.4966\text{,}$	
	$Sr.\left(\zeta_{3}\right)=0.5154\text{,}$		$Sr.\left(\zeta_{3}\right)=0.5025\text{,}$	
	$Sr.\left(\zeta_{4}\right)=0.5159$		$Sr.\left(\zeta_{4}\right)=0.5064$	

**Fig. 7:**
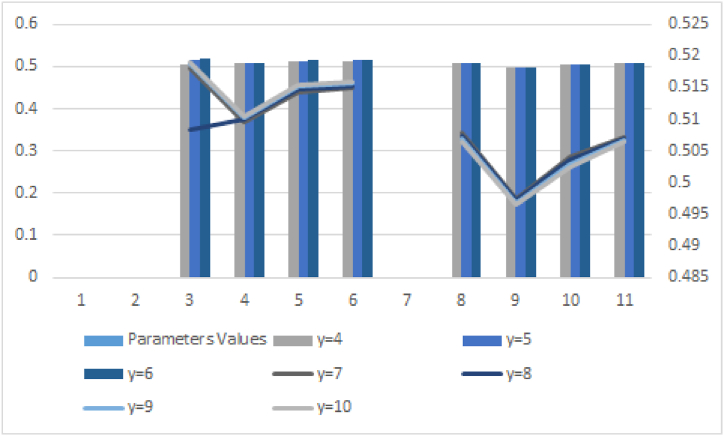
Sensitivity analysis of data given in Table 15.

From the analysis of above Table 15, we can see that as we increase the value of parameter $\prime \prime q^{\prime \prime }$ then notice the best alternative in all cases is the same which shows the authenticity of the proposed theory. Also, the graphical representation of the sensitivity analysis is given in Fig. 7.

## 9 Conclusion

The notion of the polar form of CPyFS contradicts the basic concept of the CST and FST because, in CST and FST, an element fully belongs to a set if its membership value is 1, and does not belong to a set if its membership value is zero. In this regard, the polar form of CPyFS is less applicable and non-reliable. Moreover, whenever we discuss the notion of the Cartesian form of CPyFS, then this notion is limited because whenever the decision-makers tackle the data in the form of (0.9+ι0.7,0.8+ι0.7), then the basic condition is violated. So based on these observations, both of these structures are non-reliable or limited in either case. Hence, there is a need to define such a powerful notion that can handle the limitations and non-reliability of both structures. So based on these observations, we have produced a strong and reliable notion of the Cartesian form of Cq-ROFS. Moreover, we have developed the Yager operational laws based on the Cartesian form of Cq-ROFS. We have introduced aggregation theory named Cq-ROFYWA and Cq-ROFYWG AO in Cartesian form. Based on these AOs, we have initiated a MAGDM approach to define the reliability and authenticity of the developed theory. Furthermore, we have utilized this device algorithm in the selection of temperature control systems. The comparative analysis of the proposed theory discusses the advancement of the initiated work.

The defined notions are also limited because when decision-makers need to utilize the third grade called abstinence grade along with membership grade and non-membership grade in their structure then the developed approach fails to handle such kind of situations.

We can enhance this theory to q-ROF soft entropy measures given in Ref. [54]. Moreover, we can extend these notions to some other aggregation operators like the Dombi aggregation operators proposed by Jaleel [55]. We can extend these ideas to the rough structure produced by Yi et al. [56]. More work can be done on MADM problems as given in Refs. [57,58]. We can extend these developed approaches to spherical fuzzy structure as discussed in Ref. [59] and some new MCDM approaches [60] can be discussed for the advancement of the delivered approach.

## CRediT authorship contribution statement

**Jabbar Ahmmad:** Writing – original draft, Validation, Methodology, Investigation, Conceptualization. **Tahir Mahmood:** Validation, Supervision, Resources, Project administration, Methodology, Investigation, Conceptualization. **Dragan Pamucar:** Writing – review & editing, Validation, Resources, Methodology, Investigation, Funding acquisition. **Hafiz Muhammad Waqas:** Writing – original draft, Validation, Methodology, Investigation, Conceptualization.

## Ethical statement

This study does not involve any human or animal subjects, and it is in accordance with research ethical standards.

## Data availability statement

Data will be made available on request. For requesting data, please write to the corresponding author.

## Declaration of competing interest

The authors declare that they have no known competing financial interests or personal relationships that could have appeared to influence the work reported in this paper.

The author is an AMB for this journal and was not involved in the editorial review or the decision to publish this article.
